# The significance of HAUS1 and its relationship with immune microenvironment in hepatocellular carcinoma

**DOI:** 10.7150/jca.90298

**Published:** 2024-01-16

**Authors:** Lei Tang, Zhonghuo Chen, Chao Wei, Hao Liu, Ben Wang, Taozhi Yu, Xiaofei Tao, Jiale Yang, Jiafu Guan, Jianwei Yi, Hengchang Zhu, Chen Li, Peng Tang, Kai Wang

**Affiliations:** 1 Hepatobiliary and Pancreatic Surgery Division, Department of General Surgery, the 2nd affiliated hospital, Jiangxi Medical College, Nanchang University, Nanchang 330038, China.; 2 Jiangxi Province Engineering Research Center of Hepatobiliary Disease, the 2nd affiliated hospital, Jiangxi Medical College, Nanchang University, Nanchang 330008, China.; 3 Jiangxi Province Key Laboratory of Molecular Medicine, the 2nd affiliated hospital, Jiangxi Medical College, Nanchang University, Nanchang 330008, China.; 4 Jiangxi Provincial Clinical Research Center for General Surgery Disease, the 2nd affiliated hospital, Jiangxi Medical College, Nanchang University, Nanchang 330008, China.; 5 Department of General Surgery, No. 215 Hospital of Shanxi Nuclear Industry, Xianyang 712000, China.; 6 Department of Emergency, Jiangxi Province Hospital of Integrated Chinese and Western Medicine, Nanchang 330009, China.; 7 Basic Research and Innovation Center for the Targeted Therapeutics of Solid Tumors, Ministry of Education, the 2nd affiliated hospital, Jiangxi Medical College, Nanchang University, Nanchang 330031, China.

**Keywords:** HAUS1, HCC, Immune microenvironment, Prognosis, Biomarker

## Abstract

**Background:** HAUS Augmin-like complex subunit 1(HAUS1), as a controlling gene, which affected the production of spindle was firstly discovered in Drosophila cells. Although HAUS1 has been intensively studied, but its significance and relationship with the immune microenvironment in Hepatocellular carcinoma (HCC) remain unclear.

**Materials and Methods:** All data of HCC in this paper were obtained from The Cancer Genome Atlas(TCGA), Genotype-Tissue Expression (GTEx), Gene Expression Omnibus (GEO) and the Human Protein Atlas(HPA) database. The role and potential value of HAUS1 in the tumorigenesis and development of HCC were studied by applying plenty of bioinformatics analysis methods. Knocked down the expression of HAUS1 through siRNA and further investigated the function of HAUS1 in HCC

**Results:** HAUS1 was highly expressed in HCC, which led to a poor prognosis. ROC curve analysis showed that HAUS1 had a excellent diagnostic value. It was also associated with clinical stage, pathological grade and AFP of HCC. Univariate and multivariate COX regression analysis showed that HAUS1 was an independent prognostic factor for HCC patients. HAUS1 was associated with immune cells infiltrate and immune checkpoints in HCC, and it could generate significative therapeutic results when combined with anti-CTLA4 and anti-CD274 treatment. In vitro experiments, HAUS1 was found to promote the proliferation, invasion and metastasis, participated in cell cycle regulation and inhibited apoptosis of HCC.

**Conclusion:** These results suggested that HAUS1 might serve as a potential therapeutic target, as well as a diagnostic, prognostic, and survival biomarker for HCC.

## Introduction

Hepatocellular carcinoma (HCC), which is the primary type of liver cancer, constitutes approximately 80% of all instance, of which the incidence and mortality are high. In this regard, the current research shows that liver cancer is the third culprit of cancer related mortality in recent years and liver cancer is responsible for nearly a million deaths in the whole world annually^1^-^3^. The incidence rate will exceed 1 million by 2025 on the basis of data from the World Health Organization^4^. HCC is still a serious burden on public health in China, with the incidence of fifth and the second highest mortality^5^. Hepatitis B virus, hepatitis C virus, obesity, alcoholic liver cirrhosis, smoking, iron overload, fatty liver, diabetes and exposure to various foods are certified as common risk factors for HCC^6^. Currently, Sorafenib, lenvatinib, apatinib, and bevacizumab are widely used in clinical practice for targeted HCC therapies, which can prolong the survival of patients^7^,^8^. However, due to drug resistance, tumor heterogeneity, patient intolerance and other reasons, there are still some patients who cannot benefit from the theray of these drugs, so the research and development of new drugs is imperative. The discovery of new therapeutic targets and the understanding of associated mechanisms are essential for the development of novel drugs^9^,^10^. According to current evidences, in the process of HCC occurrence and development, several genes may mutate, which cause differences in expression, affecting the growth, proliferation, intolerance and drug resistance of HCC. Therefore, these genes could serve as valuable clinical biomarkers and potential therapeutic targets for this specific population.

The HAUS1 gene encodes a protein called "HAUS Augmin-like complex subunit 1," which is a subunit of the HAUS (HepA/Unc84/Sun1) Augmin-like complex. This complex plays a role in multiple cellular processes, such as cell division and microtubule organization^11^. The Augmin complex was recorded for the first time in the formation of Drosophila S2 cell spindle thread. Subsequently, the subunit of the Augmin complex was discovered in human cells as well. Mass spectrometry identified a complete human Augmin complex consisting of eight subunits. The HAUS complex has a critical function in guaranteeing the accurate separation of chromosomes during the process of cell division (mitosis and meiosis) by facilitating the formation of microtubules and their attachment to chromosomes. Microtubules are dynamic protein filaments that play a structural role in the cell and are essential for processes such as cell division^12^,^13^. The HAUS Augmin-like complex, including HAUS1, has been found to interact with other proteins to regulate microtubule dynamics. Dysfunction of these proteins can lead to errors in cell division and may be associated with various diseases and conditions, including cancer. HAUS1 may be highly expressed in the heart and aortic tissues of a rat model of nitrate vascular resistance and may affect its resistance^14^. It is also associated with the pathogenesis of acute myeloid leukemia^15^. However, HAUS1 had rarely been studied in solid tumors, especially in HCC. So far, it is not clear whether HAUS1 can be used as a therapeutic target, whether HAUS1 in HCC affects the proliferation of hepatocellular carcinoma cells, whether it is related with immune microenvironment in HCC, whether it is involved in immune escape of HCC, whether it promotes the progression of HCC, or whether it leads to a poor prognosis. Therefore, we comprehensively studied the function and significance of HAUS1 in HCC, our findings revealed a significant upregulation of HAUS1 in HCC, and the high expression of HAUS1 reduced the survival of patients. Univariate and multivariate Cox regression analyses demonstrated that HAUS1 could independently influence the prognosis of HCC. HAUS1 has also been found to be associated with escape of immune surveillance in HCC. Its high expression promoted immune infiltrates in HCC and also interacted with immune checkpoints. Meanwhile, HAUS1 combined with immunotherapy achieved good therapeutic effect. The experiment also demonstrated that HAUS1 regulated the cell cycle, promoted proliferation, inhibited apoptosis, and enhanced the invasion and metastasis ability of hepatoma carcinoma cells. These findings may lead to new insights of diagnosis, prognosis and therapy HCC in this population.

## Materials and Methods

### The Cancer Gene Atlas (TCGA) database

The Cancer Gene Atlas database (https://www.cancer.gov/) is a part of the common cancer genome project, providing more than 20000 genomic sequences, epigenomic, methylation, transcriptomic and copy number data for more than 33 different types of cancers. Common research hopes to improve diagnostic methods, nursing standards and provide new methods for cancer prevention^16^,^17^. The HCC sample data was downloaded from the TCGA database. Each sample undergoing multiple pathological tests confirming HCC, adjacent tissues showed no tumor cells and exhibited characteristics consistent with normal liver cells^18^. Analysis of HCC and matched normal samples revealed differential gene expression, the differential expression genes (DEG) were obtained and the significance of these DEGs to HCC was evaluated, dentifying high expression of HAUS1 in HCC with a significant impact on therapy and prognosis.

### Gene Expression Profiling Interactive Analysis (GEPIA) database

The GEPIA (http://gepia.cancer-pku.cn/) database as a fast and customizable online analysis tool based on TCGA and GTEx databases and provides some analytical methods including differential expression analysis, correlation analysis, mapping, similar gene testing, patient survival analysis and dimensional drop analysis^19^. We analyzed the expression and evaluated their meanings on patient survival, prognosis and correlation molecules of eight subunits of the Augmin complex through the GEPIA database and established a significantly related molecule with cancers.

### Tumor Immune Estimation Resource (TIMER) 2.0 database

The TIMER2. 0 (http: //timer. cistrome. org/) database provides four modules to study immune infiltrates. TIMER2. 0 web servers support comprehensive analysis and visualization of immune infiltrates for various cancer^20^. We analyzed the relationship between HAUS1 and immune cell infiltration in HCC using the TIMER2.0 database, while also investigating the relationship between HAUS1 and immune checkpoint molecules CTLA-4, PD-1 (PDCD1), and PD-L1 (CD274).

### The Human Protein Atlas (HPA) database

The HPA (http: //www. proteinatlas. org) database is an excellent human protein drawing project used antibody based on imaging, brainstem, transcriptology, mass spectrometry-based proteomics, and so on to draw all human proteins. It includes IHC analysis of a number of protein coding genes in different cancer patients using various techniques^21^,^22^. From the HPA database, the IHC images of HAUS1 were downloaded including liver cancer and normal liver tissues to identify the differences.

### Kaplan-Meier plotter database

Kaplan-Meier plotter (kmplot.com) database, as a survival analysis tool with data mainly from GEO, EGA and TCGA databases, can evaluate gene samples of multiple tumors and study the relationship between a gene and tumor survival, assess its ability as a survival biomarker^23^. By analyzing the effect of HAUS1 on the OS (overall survival), RFS (recurrence-free survival), PFS (progression-free survival) and DSS (disease-specific survival) of HCC, we determined that HAUS1 can be used as a survival biomarker.

### Cell culture and SiRNA transfection

The human hepatoma cell lines were used in this study including MHCC-97H, HepG2, HCCLM3, Hep3B, Huh-7, and normal hepatocyte 7702, from Biochemistry and Cell Biology, Chinese Academy of Sciences (Shanghai). Cultured at least twice before transfection. SiRNA's mimics were obtained from RiboBio (Guangzhou, China). On the basis of the maker's explanation, SiRNA transfection using riboFECT™CP (RiboBio). Western blot were performed for verification^24^. The sequence of siRNA is listed below: siHAUS1-1: TGGAGATCATCCTA TTCCA, siHAUS1-2: GGATGTCTACCTGGTAATA, siHAUS1-3: CACGGACCACAGAGATTTT.

### Western blot

Western blot was used to extract and quantify total protein according to the general method. After electrophoresis, protein samples were transferred onto PVDF films under constant current of 260 mA. The membrane was closed for 1 to 3 hours at 5% degreased milk at room temperature, and then the target protein binding of primary antibody was performed at 4 ℃ overnight, secondary antibodies at room temperature. TBST washes the membrane three times, ten minutes at a time. The membrane was visualized and analyzed using image lab software^25^.

### CCK-8 assay

According to the protocol's description, getting 1000 cells and planting them into each well of the 96-well plates, leaving blank controls. CCK-8 reagent (Cat#: K1018; APExBIO, China) were added once a day for 4 consecutive days after cell culture, and the OD value was measured with the absorbance of 450 nm after 37 °C incubation^26^.

### EdU assay

The treated cells were planted in 96-well plates and cultured overnight until the cells adhered to the wall to grow. According to the protocol (Cat#: K1175; APExBIO, China), the cells were stained, and then observed on the fluorescence microscope, the nuclei were stained blue and the proliferated cells were stained green.

### Flow cytometry

According to the protocols, when detecting cell cycle (Cat#: KGA512; KEYGEN Bio, China), cells were cleaned and fixed overnight with 75% ethanol chilled in advance, then stained and detected on flow cytometry. When detecting apoptosis (Cat#: KGA108; KEYGEN Bio, China), cells can be stained and detected after cleaning, and the detection should be completed within 1 hour at the latest.

### Wound healing assay

Three horizontal lines are uniformly drawn Under the 6 well plate, the width is 0. 5-1cm. The cells were cultured using complete culture medium and planted in 6-well plates until cell density reached 80-90% Using the pipette tip, vertical lines are drawn vertically across the board. The cells in the 6-well plates were washed three times with PBS, and culture was continued by adding 2 ml serum free medium at 37 °C. Taking pictures at 0, 24 and 48 hours respectively^27^.

### Transwell assay

Taking 200ul cells with a density of 2 × 10^5^ cells / ml, which were suspended by serum-free medium, and then it was added to the upper chamber of the uncoated or matrigel-coated Transwell chamber (BD Biosciences). Add 500ul of complete medium to the lower chamber. After 36 hours of cultivation, carefully remove untransferred cells on the membrane surface by swabs, fixed cell. After 0. 1% Crystal Purple staining, the non-migrated cell on the membrane upper surface was carefully wiped off with cotton swabs, then took pictures^28^.

### Data analysis

Data analysis used R v3. 6. 1 (https: //www. r-project. org/), R v4. 2. 1, GraphPad Prism 9. 0. 0(http: //www. graphpad-prism. cn) and SPSS 26. 0 (SPSS Inc, Chicago, IL). It takes an average value ± standard difference of three independent experiments. The student t-test analyzes the differences between two groups. For two or more groups, the unidirectional ANOVA would be used. More, in the analysis of univariate and multivariate analysis, using the logistic regression model to prove the independent prognostic biomarker. P value < 0. 05 is statistically significant.

## Results

### HAUS1 was highly expressed in HCC

**Figure 1:**
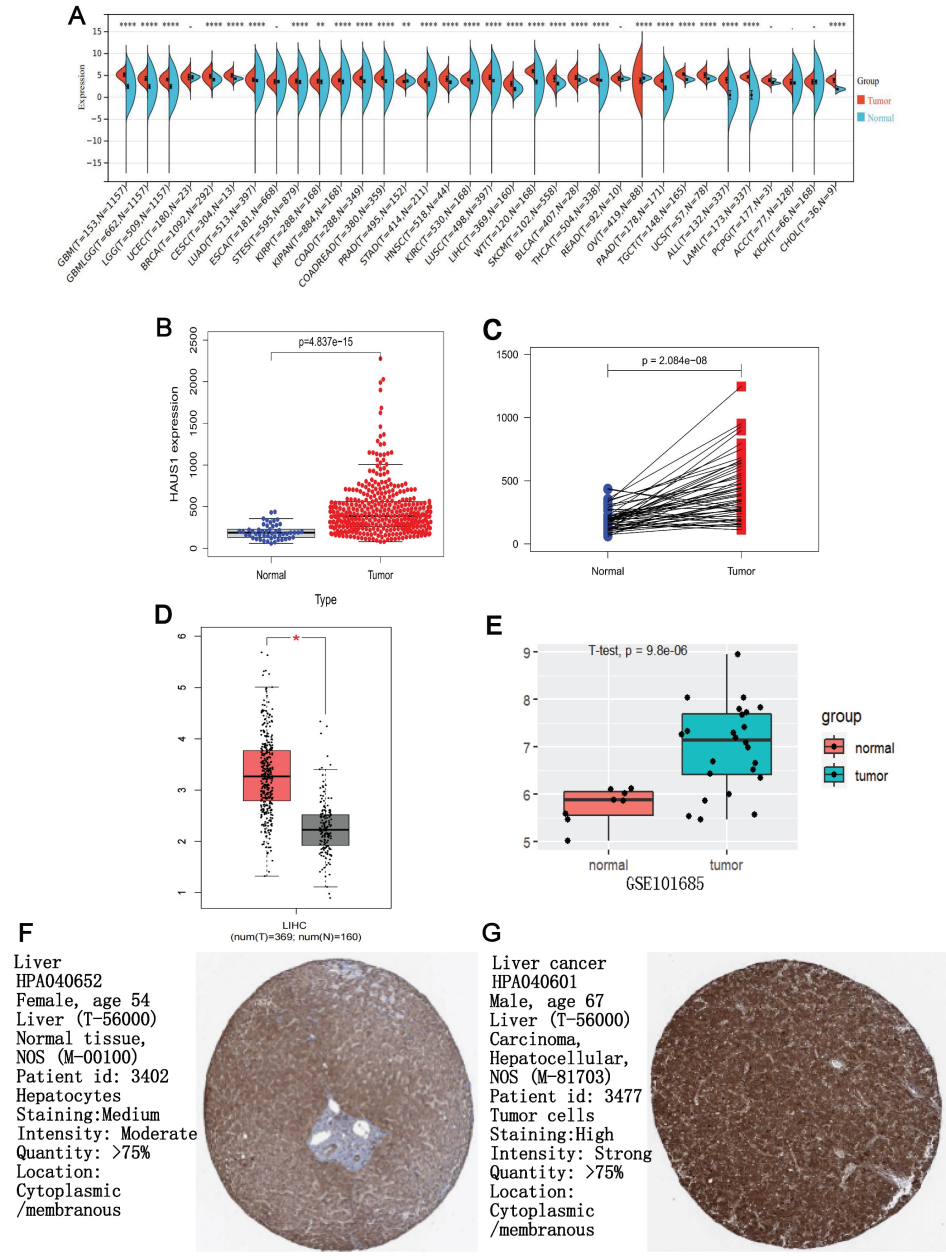
(A) HAUS1 expression in different tumors, (B-C) Through TCGA database and R language, HAUS1 was highly expressed in paired and unpaired samples of HCC. (D) HAUS1 was highly expressed in HCC through GEPIA database. (E) HAUS1 was highly expressed in HCC through GSE101685 database. (F-G) The immunohistochemical map of HPA database showed that HAUS1 was Highly expressed in HCC. *, P<0.05; **, P<0.01; ***, P<0.001; ****, P<0.0001; ns, no significance.

We used sangerbox3.0 (sangerbox.com) to research the mRNA expression level of HAUS1 gene in 34 tumors, and the data came from TCGA and GTEx databases, It was found that HAUS1 was only low expressed in PRAD and OV tumors. In the rest of the tumor except UCEC, ESCA, READ, PCPG, ACC, KICH all were highly expressed, especially in HCC (figure 1 A). Subsequently, we separately analyzed the expression of HAUS1 in HCC and paired and unpaired samples using the TCGA database, and found that the expression of HAUS1 increased in LIHC (Liver hepatocellular carcinoma) (Figure 1B and 1C). The GEPIA and GSE101685 databases both confirmed the high expression of HAUS1 in HCC (Figures 1D and 1E). Immunohistochemical maps of HPA database also found that HAUS1 was significantly highly expressed in HCC (Figures 1F and 1G).

### High expression of HAUS1 led to a poor prognosis for HCC

**Figure 2:**
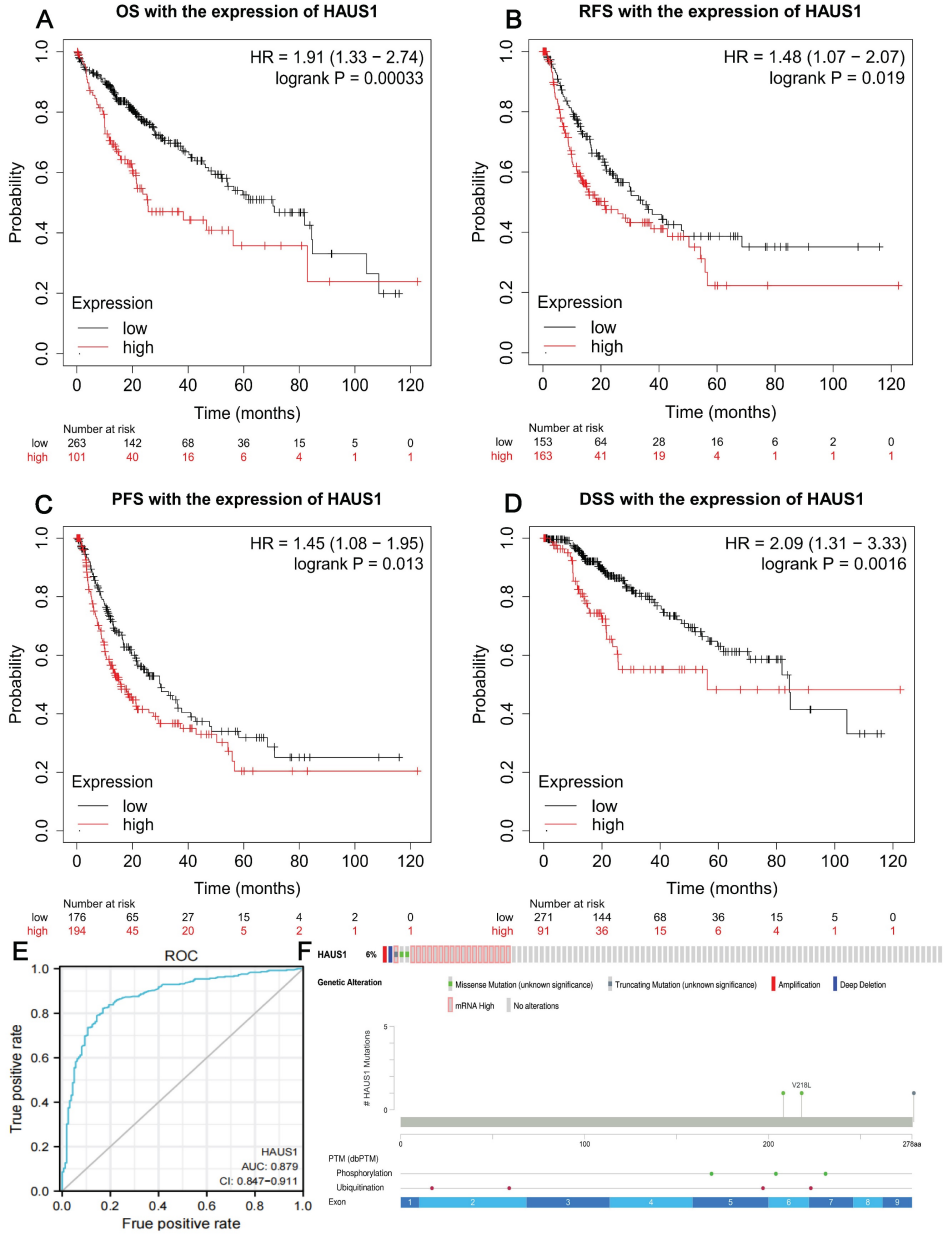
(A-D) it was found that high expression of HAUS1 all reduced OS. RFS, PFS, and DSS, leaded to a poor prognosis according to the Kalan-Meier plotter database. (E) ROC curve analysis found that HAUS1 had a very high diagnostic value for HCC. (F) HAUS1 was mutated and modified in the patients with HCC.

Through the Kalan-Meier plotter database, we found that when HAUS1 is highly expressed, the OS, RPS, PFS and DSS of HCC patients are significantly reduced, and the survival period is greatly shortened, indicating that the high expression of HAUS1 in HCC would lead to a poor prognosis (Figure 2A-2D). It indicated that HAUS1 could be used as a survival biomarker.

### The diagnostic value of HAUS1, its mutation and modification in HCC

By analyzing the ROC diagnostic curve of HAUS1 in HCC, it was found that the area under the ROC curve was 0.879, indicating that HAUS1 had a very high diagnostic value on HCC (Figure 2E). At the same time, we found that 6% of 360 HCC patients had mutations in HAUS1 through the cBioPortal database (www.cbioportal. org), Among them, missense mutation and high mRNA mutation are the most common. During translation, HAUS1 may be modified by phosphorylation and ubiquitination (Figure 2F).

### Clinical features and univariate and multivariate COX regression analysis of HAUS1 in HCC

Clinical feature analysis related to HCC showed significant correlation between HAUS1 expression and age, race, histopathology and AFP in patients (Table 1). At the same time, Cox regression analysis identified several factors that significantly influence the survival of HCC patients, including T stage, M stage, histopathology stage, tumor status, and HAUS1 expression. In multivariate analysis, only tumor state and HAUS1 expression are important predictors of patient survival, So HAUS1 could serve as a superb independent prognostic factor for HCC (Table 2).

### Relationship between HAUS1 and immune microenvironment in HCC

**Figure 3:**
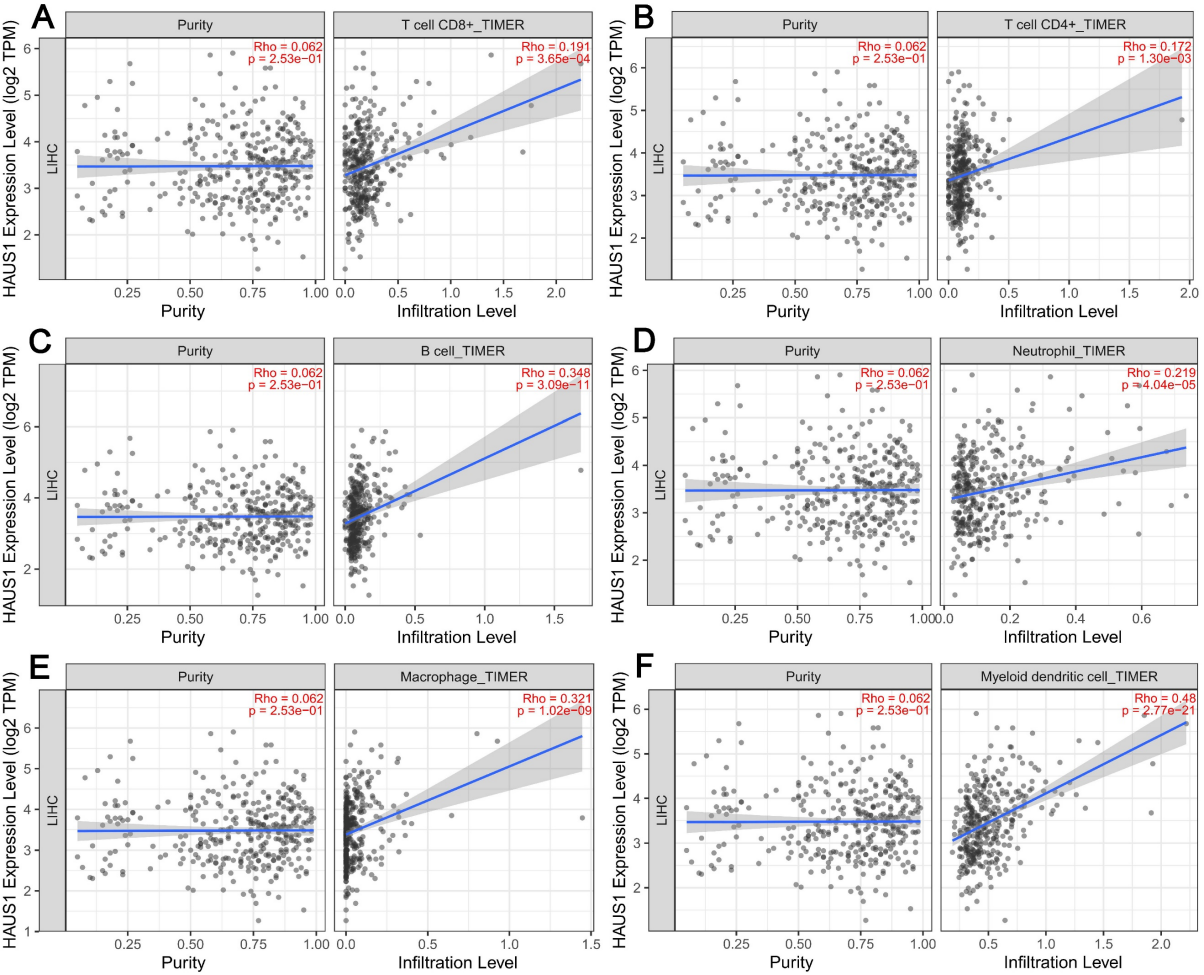
(A-F) HAUS1 was significantly correlated with the infiltrate levels of six key kinds of immune cells including T cells CD4+, T cells CD8+, B cells, Neutrophils, Macrophages and Myeloid dendritic cells in HCC.

The emergence and growth of HCC was closely related to the immune microenvironment, so we speculated that HAUS1 may be related to the immune microenvironment. Therefore, in order to research the relationship between HAUS1 and immune microenvironment in HCC and study its mechanism, TIMER2.0 database was adopted, revealing that HAUS1 was significantly associated with T cells CD4 +, T cells CD8 +, B cells, Neutrophils, Macrophages and Myeloid dendritic cells (Figure 3A - 3F). Immune cells were significantly enriched in tumor tissues with high HAUS1 expression, and infiltration was also increased. In HCC tumor tissues with high HAUS1 expression, the immune microenvironment might shift from activated effector cells to quiescent immune inhibitory cells. These results suggested that HAUS1 might promote the emergence and growth of HCC by influencing the infiltrates of immune cells.

### The relationship between HASU1 and immune checkpoints in HCC

**Figure 4:**
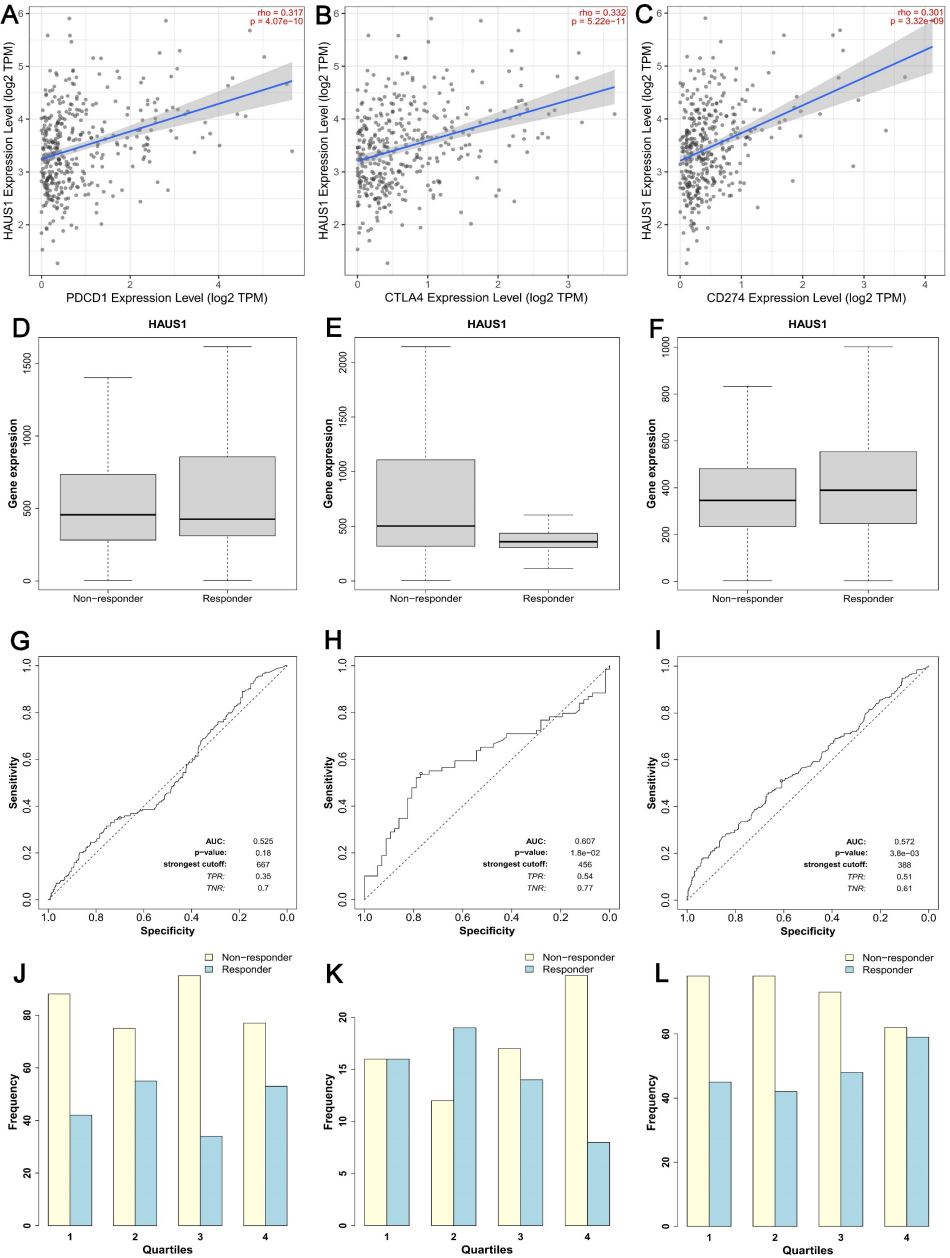
(A-C) HAUS1 was positively correlated with immune checkpoints PDCD1, CTLA4, and CD274. (D-L) HAUS1 had significant therapeutic efficacy when combined with anti-CTLA4 and CD274, but not when combined with PDCD1.

CTLA-4、PDCD1 and CD274 are three crucial immune checkpoints that play significant roles in immune surveillance and immune escape within tumors. Through TIMER2.0 database, we found that HAUS1 was significantly positively correlated with PDCD1, CTLA4 and CD274 in HCC (Figure 4A-4C). The high correlation between HAUS1 and CTLA-4, PD-1 (PDCD1) and PD-L1 (CD274) suggested that high expression of HAUS1 might facilitate HCC to escape immune cell recognition through immune checkpoints and thus avoid being attacked. Meanwhile, according to the Kalan-Meier plotter database, HAUS1 combined with anti-CTLA4 and anti-CD274 could improve therapeutic effects for patients with HCC. Although HAUS1 combined with PDCD1 has no significant therapeutic effect, it still was still referential (Figure 4D-4L). These results indicated that HAUS1 might influence HCC progression through immune escape and could be used as a therapeutic target for drugs.

### Protein-protein interaction network map of HAUS1-related genes, GO and KEGG enrichment analysis

**Figure 5:**
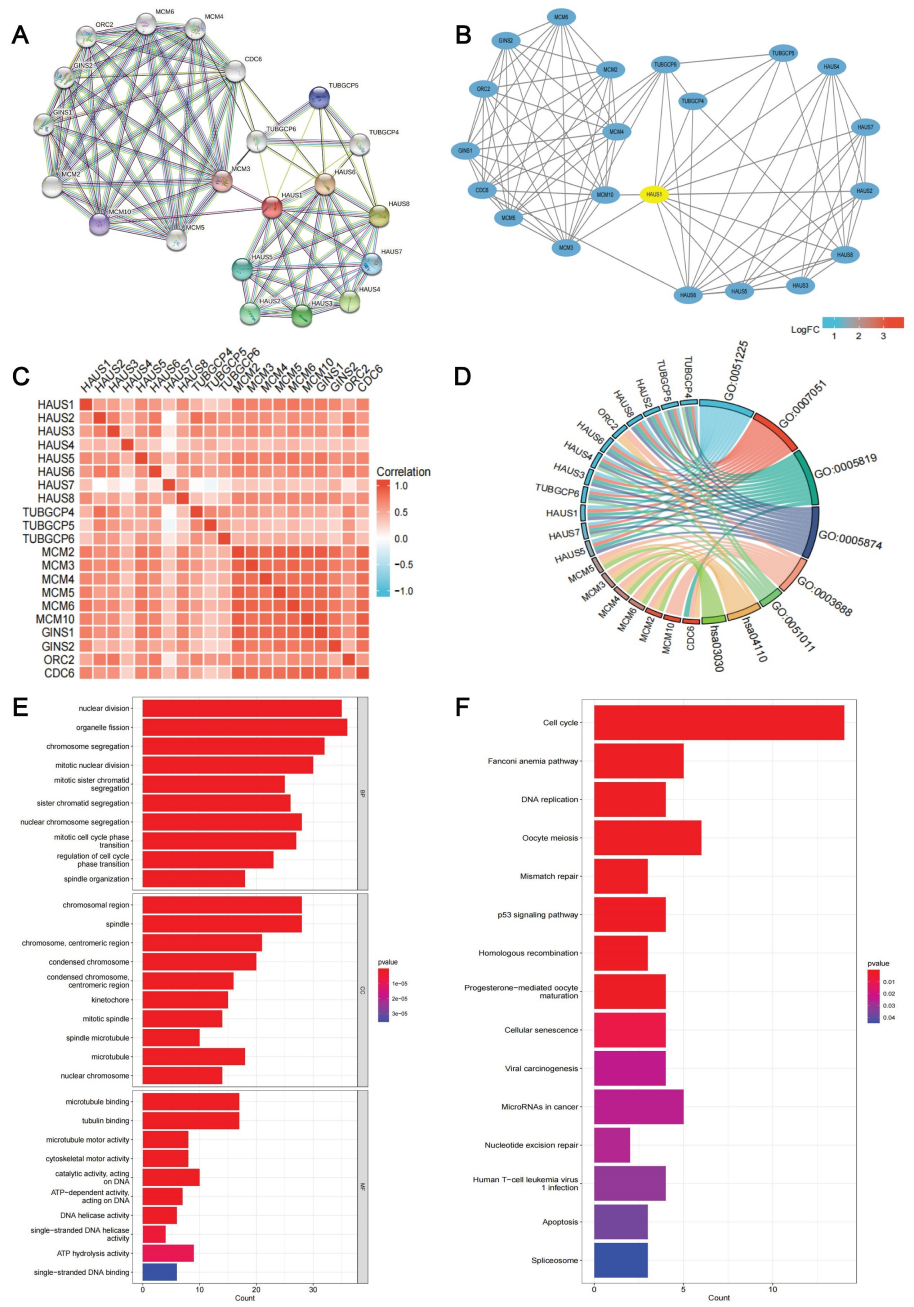
(A-B) PPI network of upstream and downstream genes associated with HAUS1 and visualization. (C) Correlation of 20 genes on PPI network. (D) GO and KEGG analysis of 20 genes in PPI network. (E-F) GO and KEGG analysis of top 100 genes associated with HAUS1.

In order to explore the function of HAUS1, we searched the upstream and downstream genes of HAUS1 through the STRING (string-db. org) database (Figure 5A) and visualized the PPI network by using Cytoscape3.9.1 (Figure 5B). Moreover, their correlation was analyzed, and a significant correlation was found (Figure 5C). The GO and KEGG enrichment analysis were carried out. But there are too few genes to fully demonstrate its function (Figure 5D). Subsequently, 100 HAUS1-related genes were obtained using the GEPIA database, and GO and KEGG enrichment analysis were executed. According to the GO analysis,we found that nuclear division, organelle fission, chromosomal region, spindle, microtubule binding, and tubulin binding are the most common part of cellular components, molecular functions and biological processes (Figure 5E).

During KEGG enrichment analysis, cell cycle, Fanconi anemia pathway and DNA replication were the most common pathways for related molecules (Figure 5F). In short, these genes were found to be related to cell proliferation and cell cycle.

### HAUS1 promoted the proliferation, migration and invasion of HCC

**Figure 6:**
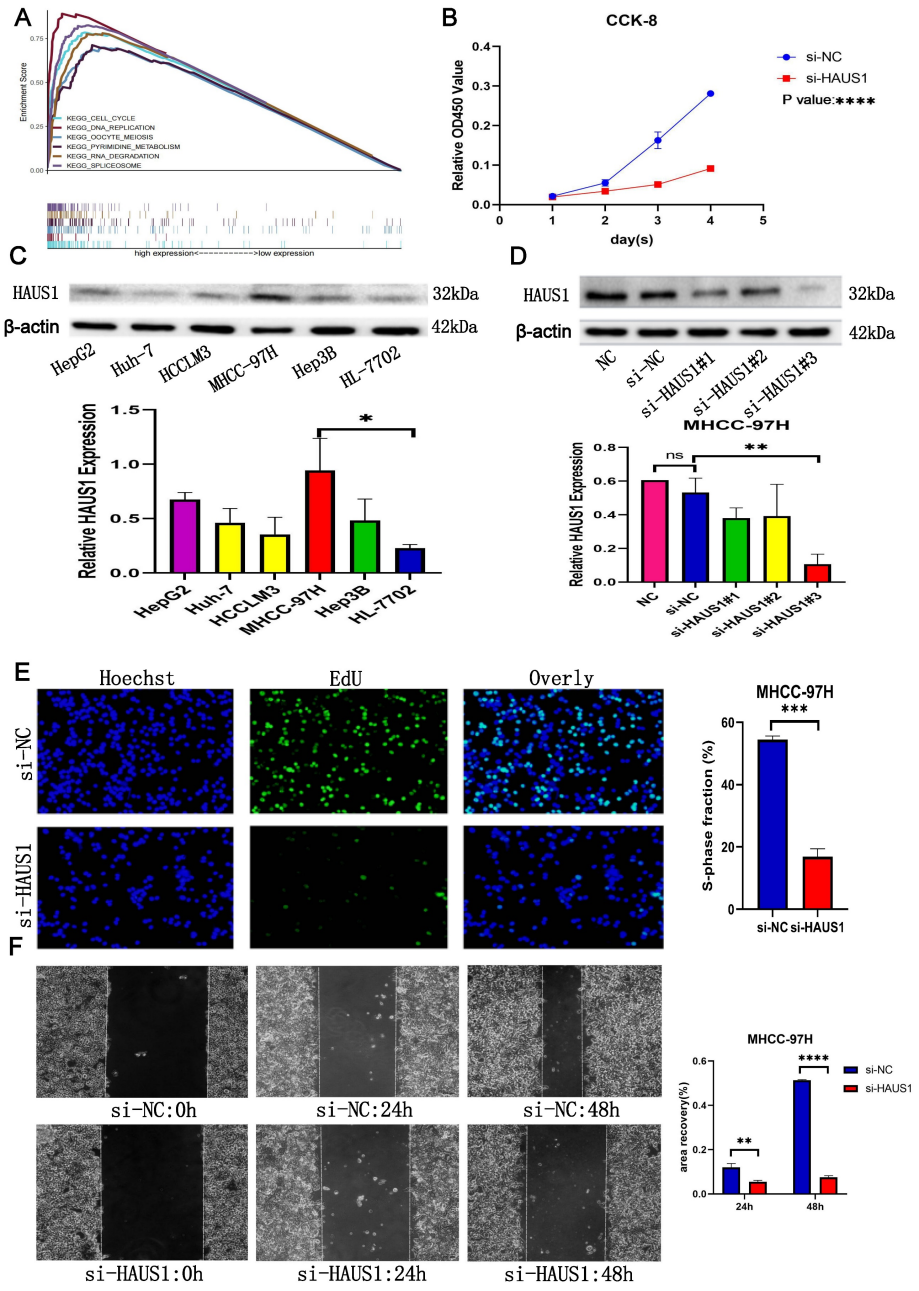
(A) The top six pathways associated with high expression of HAUS1. (B) CCK-8 analysis of proliferation capacity. (C) HAUS1 expression in hepatoma carcinoma cells and normal hepatocyte and histogram. (D) siRNA interference efficiency and histogram. (E) EdU assay: green fluorescence means cells were proliferating. (F) Normal group and interference group Wound Healing Assay results.

**Figure 7:**
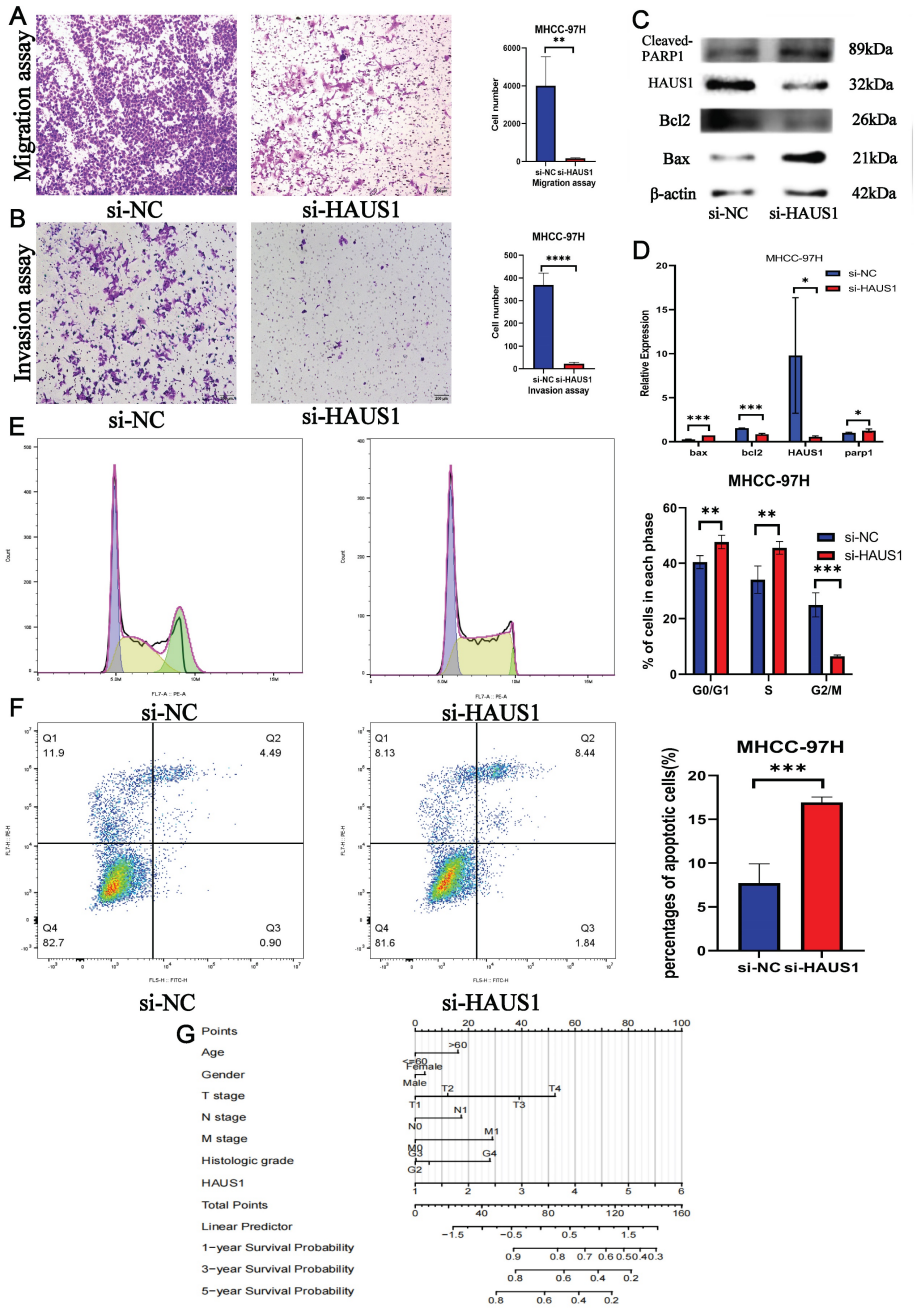
(A) Migration assay. (B)Invasion assay. (C-D) Interference with HAUS1 expression revealed changes in proteins associated with apoptosis. (E) Most cells were blocked in the G0/G1 and S phases. (F) The number of apoptotic cells increased. (G) HAUS1-related nomogram model.

By means of bioinformatics analysis, we found the top 6 pathways associated with high HAUS1 expression effects were related to cell cycle, growth and proliferation (Figure 6A), which revealed that HAUS1 might promote the growth of HCC. Subsequently, we intended to prove our conjecture experimentally. At the protein level, the expression of HAUS1 was higher in hepatoma carcinoma cells, and MHCC-97H cells with the highest expression were selected for study (Figure 6C). Western blot experiment proved that si-HAUS1#3 interfered with the expression of HAUS1 with the strongest effect (Figure 6D). si-NC was the normal group, and si-HAUS1 was the treatment group. The growth of MHCC-97H cells after HAUS1 interference was significantly decreased by CCK-8 and EdU assay (Figure 6B and 6E). By the Wound Healing assay, the migration ability of MHCC-97H cells in the treatment group was significantly decreased at 24h and 48h, which proved that interfere with the expression of HAUS1 could inhibit the migration ability of MHCC-97H cells (Figure 6F). This finding was also proved by the transwell assay (Figure 7A). Moreover, HAUS1 interference suppresses the invasion ability of cancer cells (Figure 7B).

### HAUS1 regulated cell cycle and inhibited apoptosis of HCC

By flow cytometry, we found that most hepatoma carcinoma cells were blocked in G0/G1 and S phase when HAUS1 expression was interfered, while cells in G2/M phase were significantly reduced (Figure 7E). At the same time, the number of hepatoma carcinoma cells apoptosis also increased significantly (Figure 7F). WB experiment also proved that HAUS1 could inhibit the apoptosis of hepatoma cells (Figure 7C). Finally, HAUS1 was further used to establish a nomogram model to evaluate the prognosis of HCC patients (Figure 7G).

## Discussion

Hepatocellular carcinoma (HCC) is the largest component of primary liver cancer and causes nearly one million deaths worldwide each year and the highest incidence in Asia and Africa^29^. Considering that early symptoms of HCC is not clear, most patients have progressed to the end before being detected^30^. This particular patient population is slightly benefited from currently available treatments and usually dies three months after the disease diagnosis^31^. Hence, the identification of new and meaningful diagnostic biomarkers and therapeutic targets for early-stage HCC is of utmost importance^32^. HAUS1 is significant for the spindle assembly as the first subunit of the Human Augmin Complex and some researchers have found that HAUS1 and HAUS6 are similar, interact with NEDD1, target spindle microtubules^33^, participate in forming cellular spindle filaments, cell mitosis, and regulating cell cycles^34^. Current evidence suggested that HAUS1 was closely associated with tumor formation and might contribute to tumor progression^35^. Analysis of TCGA, GTEx, GEO and HPA databases showed that HAUS1 was significantly highly expressed in HCC, which was associated with a worse prognosis and reduced survival among patients. It could be a survival biomarker. ROC curve analysis showed that HAUS1 had a very high diagnostic significance for HCC. Univariate and multivariate COX regression analysis also confirmed that HAUS1 could independently affect the prognosis of HCC and was correlated with clinical features. High expression of HAUS1 was associated with higher TMN staging of HCC. These findings suggested that HAUS1 had the potential to serve as a new diagnostic biomarker for HCC. However, the possible ways in which HAUS1 regulated HCC progression remain unknown.

Tumor progression and immunomodulation are shown to be inseparable^36^. We suspected that HAUS1 may effect the pathogenesis and development of HCC through immune microenvironment. Subsequent analysis confirmed that HAUS1 was positively correlated with the infiltrates level of immune cells. At the same time, HAUS1 was also positively correlated with PDCD1, CTLA4 and CD274. This suggested that the high expression of HAUS1 might lead to immunosuppression by influencing the composition of immune cells and the level of immune infiltration, while also regulating immune checkpoints effect on the recognition of immune cells, allowing HCC to evade immune cell attacks. HAUS1 might regulate the tumorigenesis of HCC through immune microenvironment and promoted the deterioration of HCC. Besides, HAUS1 combined with anti-CTLA4 and anti-CD274 therapy showed significant therapeutic effect, suggesting that HAUS1 could be one of the therapeutic targets. Subsequently, we demonstrated through experiments that HAUS1 was involved in regulating cell cycle and inhibiting apoptosis. Interference with the expression of HAUS1 would block the cell cycle in G0/G1 and S phases, and increase the apoptosis of hepatocellular carcinoma cells. At the same time, the ability of proliferation, metastasis and invasion of hepatoma carcinoma cells decreased significantly. These results indicated that HAUS1 enhanced the proliferation, invasion and metastasis of HCC and reduced the survival time of patients. Although our research has evaluated the significance of HAUS1 in HCC from the aspects of immune microenvironment, growth, prognosis, etc, but there are still some deficiencies, and the mechanism of HAUS1 affecting the development of hepatocellular carcinoma cells needs to be further studied.

## Conclusion

In conclusion, HAUS1 combined with immunotherapy achieved significative results and the high expression of HAUS1 promoted HCC tumorigenesis and development by regulating immune microenvironment, regulating cell cycle, inhibiting apoptosis and other ways, leading to poor prognosis, which may have important diagnostic, prognostic and therapeutic value. In the future, HAUS1 maybe a candidate therapeutic target, a survival, diagnostic and prognostic biomarker for HCC patients.

## Supplementary Material

Supplementary information.

## Figures and Tables

**Table 1:** The correlation between HASU1 expression and clinicopathological features for HCC patients.

Characteristic		Low expression of HAUS1 (n=187)	High expression of HAUS1 (n=187)	p	statistic	method
Gender, n (%)	Female	62 (16.6%)	59 (15.8%)	0.825	0.05	Chisq.test
	Male	125 (33.4%)	128 (34.2%)			
Age, n (%)	<=60	78 (20.9%)	99 (26.5%)	**0.034**	4.51	Chisq.test
	>60	109 (29.2%)	87 (23.3%)			
BMl, n (%)	<=25	84 (24.9%)	93 (27.6%)	0.415	0.66	Chisq.test
	>25	84 (24.9%)	76 (22.6%)			
Race, n (%)	Asian	67 (18.5%)	93 (25.7%)	**0.010**	9.3	Chisq.test
	Black orAfricanAmerican	7 (1.9%)	10 (2.8%)			
	White	107 (29.6%)	78 (21.5%)			
T stage, n (%)	T1	101 (27.2%)	82 (22.1%)	**0.249**	4.12	Chisq.test
	T2	42 (11.3%)	53 (14.3%)			
	T3	36 (9.7%)	44 (11.9%)			
	T4	6 (1.6%)	7 (1.9%)			
N stage, n (%)	NO	127 (49.2%)	127 (49.2%)	1.000		Fsher.test
	N1	2 (0.8%)	2 (0.8%)			
M stage, n (%)	MO	130 (47.8%)	138 (50.7%)	0.361		Fisher.test
	M1	3 (1.1%)	1 (0.4%)			
Pathologic stage, n (%)	Stage I	94 (26.9%)	79 (22.6%)	0.152		Fisher.test
	Stage Il	39 (11.1%)	48 (13.7%)			
	Stage III	37 (10.6%)	48 (13.7%)			
	Stage IV	4 (1.1%)	1 (0.3%)			
Histologic grade, n (%)	G1	39 (10.6%)	16 (4.3%)	**<0.001**	19.78	Chisq.test
	G2	92 (24.9%)	86 (23.3%)			
	G3	50 (13.6%)	74 (20.1%)			
	G4	2 (0.5%)	10 (2.7%)			
AFP (ng/ml), n (%)	<=400	125 (44.6%)	90 (32.1%)	**<0.001**	12.33	Chisq.test
	>400	21 (7.5%)	44 (15.7%)			

**Table 2:** The univariate and multivariate COX regression analysis of the prognosis of HCC patients

Variables (n)		Total (N)	HR (95% CI)Univariate analysis	P valueUnivariate analysis	HR (95% CI)Multivariate analysis	P valueMultivariate analysis
Age (373)	<=60	177	1.205 (0.850-1.708)	0.295		
	>60	196				
Gender (373)	Female	121	0.793 (0.557-1.130)	0.2		
	Male	252				
T stage (370)	T1	183	2.126 (1.481-3.052)	**<0.001**	0.929 (0.126-6.828)	0.942
	T2&T3&T4	187				
N stage (258)	NO	254	2.029 (0.497-8.281)	0.324		
	N1	4				
M stage (272)	M0	268	4.077 (1.281-12.973)	**0.017**	2.457 (0.572-10.559)	0.227
	M1	4				
Pathologic stage (349)	Stage I	173	2.090 (1.429-3.055)	**<0.001**	2.397 (0.315-18.221)	0.398
	Stage ll & Stage II & Stage IV	176				
Tumor status (354)	Tumor-free	202	2.317 (1.590-3.376)	**<0.001**	1.916 (1.199-3.060)	**0.007**
	With tumor	152				
Histologic grade (368)	G1	55	1.188 (0.721-1.958)	0.499		
	G2&G3&G4	313				
HAUS1 (373)	LOW	187	1.594 (1.126-2.257)	**0.009**	**1.873 (1.182-2.967)**	**0.008**
	High	186				
