# Supplementary figures and images for: The significance of HAUS1 and its relationship with immune microenvironment in hepatocellular carcinoma
Source: J Cancer. 2024 Jan 16;15(5):1328–41. doi: 10.7150/jca.90298 (PMC10861820; doi:10.7150/jca.90298)

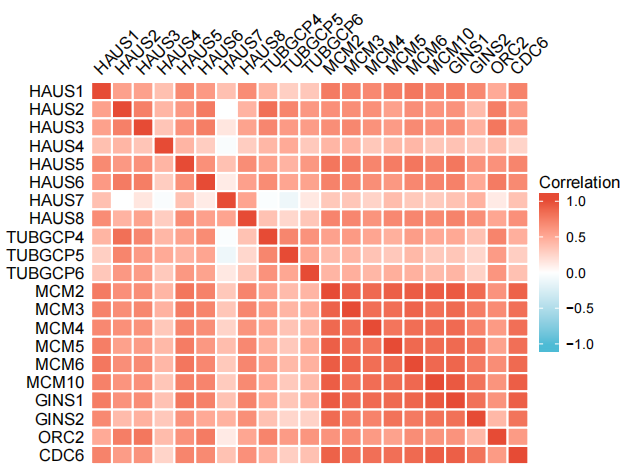

Supplement: Supplementary file 1 — Supplementary information. [file jcav15p1328s1.zip › Images Based on Data Mining and Bioinformatics Methods/Correlation heat map2022-11-01_18_21_23_00.png]

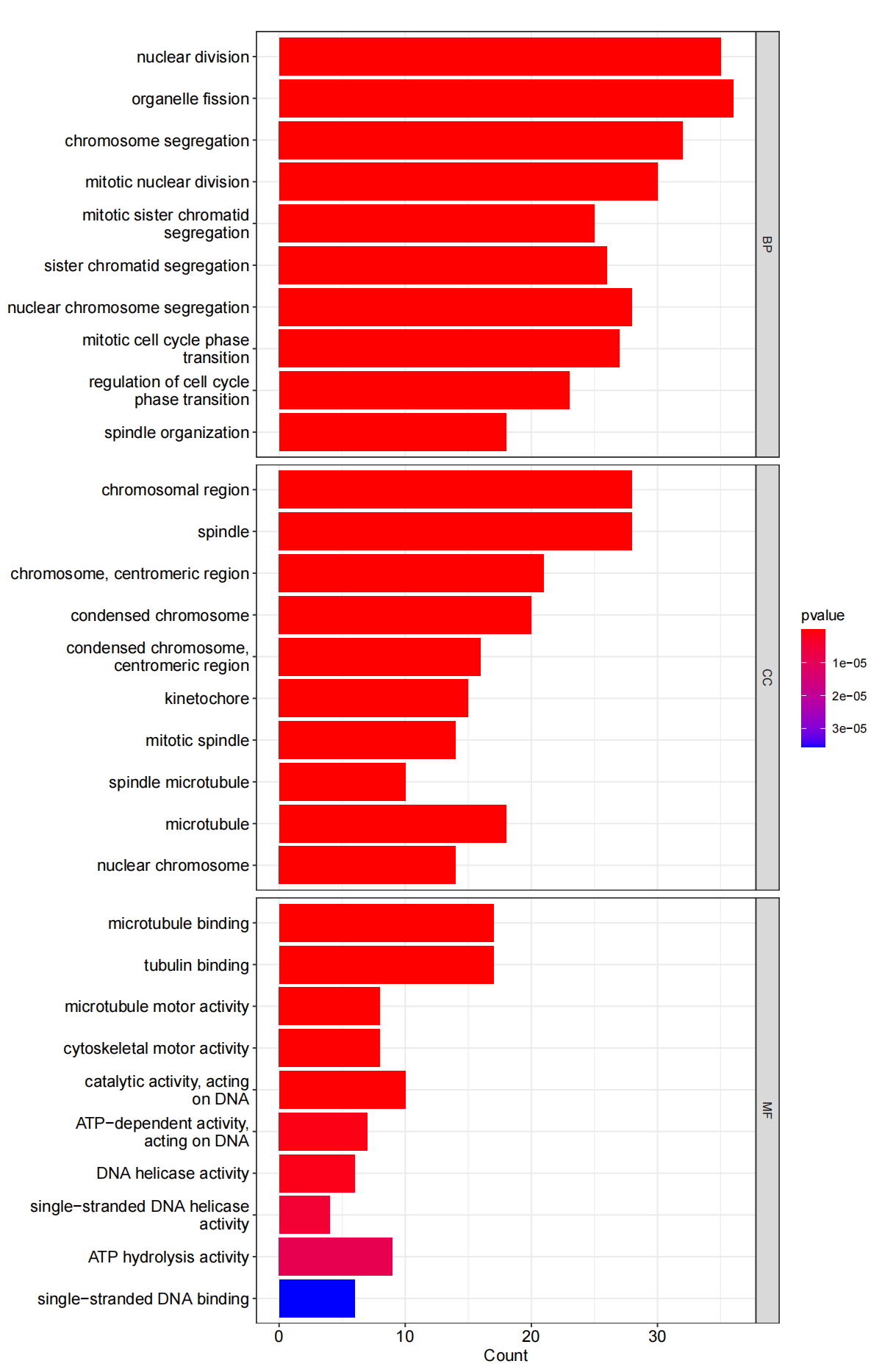

Supplement: Supplementary file 1 — Supplementary information. [file jcav15p1328s1.zip › Images Based on Data Mining and Bioinformatics Methods/go.png]

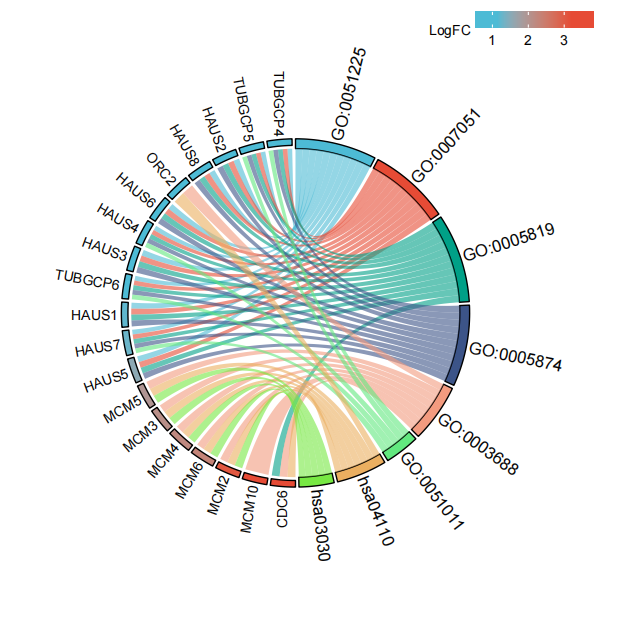

Supplement: Supplementary file 1 — Supplementary information. [file jcav15p1328s1.zip › Images Based on Data Mining and Bioinformatics Methods/GOKEGG弦图_2022-11-01_19_40_44_00.png]

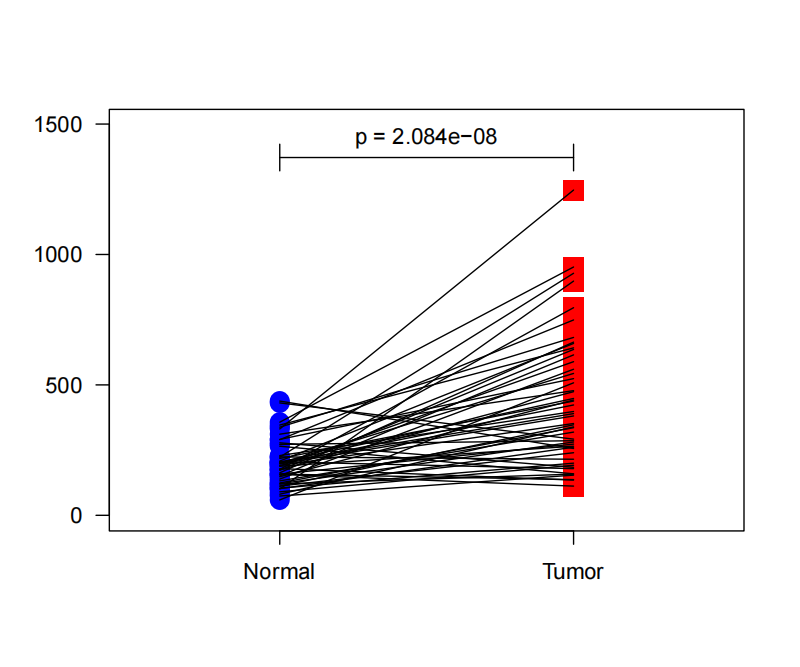

Supplement: Supplementary file 1 — Supplementary information. [file jcav15p1328s1.zip › Images Based on Data Mining and Bioinformatics Methods/HAUS1 Paired_00.png]

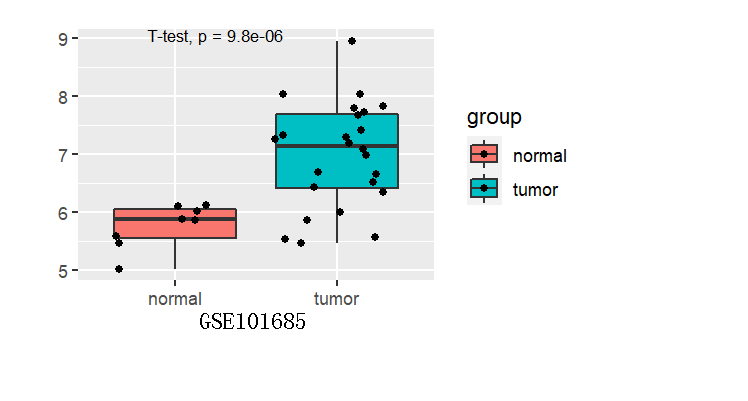

Supplement: Supplementary file 1 — Supplementary information. [file jcav15p1328s1.zip › Images Based on Data Mining and Bioinformatics Methods/HAUS1.png]

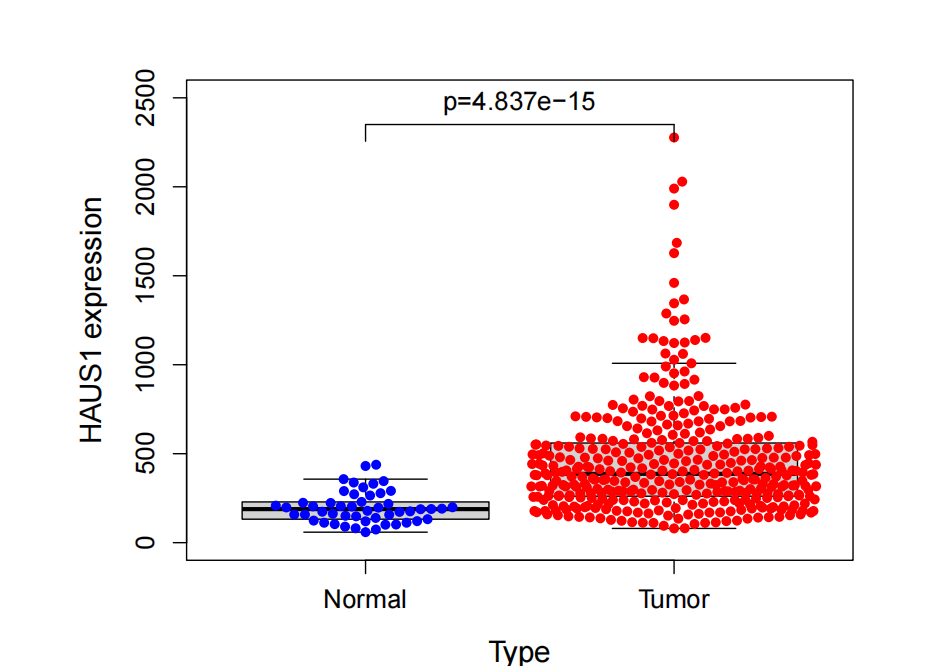

Supplement: Supplementary file 1 — Supplementary information. [file jcav15p1328s1.zip › Images Based on Data Mining and Bioinformatics Methods/HAUS1_00.png]

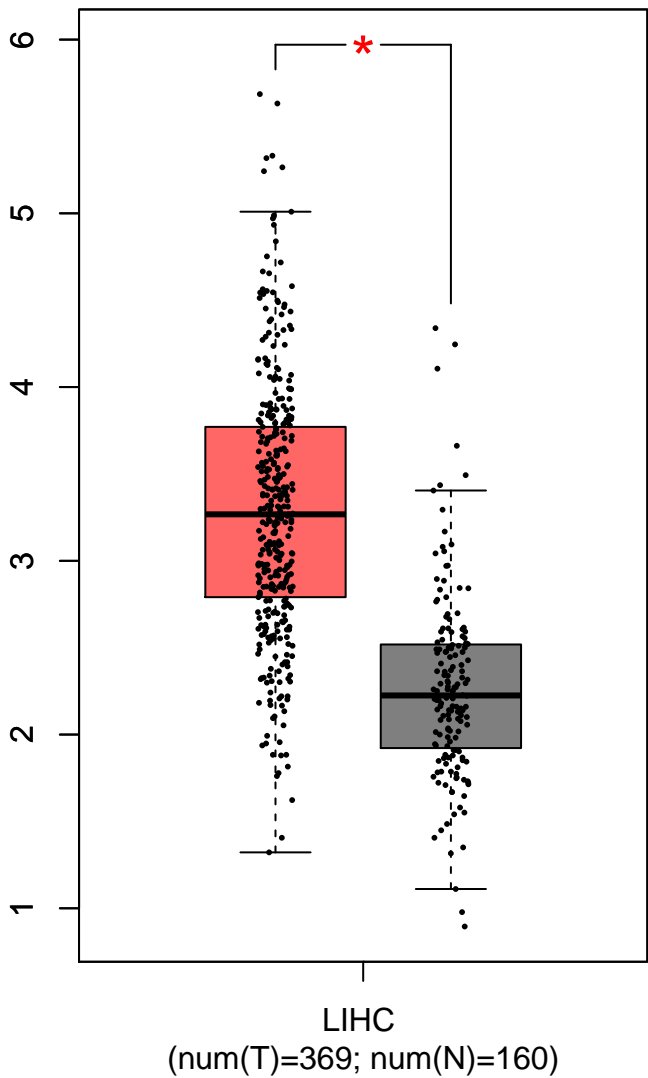

Supplement: Supplementary file 1 — Supplementary information. [file jcav15p1328s1.zip › Images Based on Data Mining and Bioinformatics Methods/HAUS1_boxplot_0FPFo.pdf]

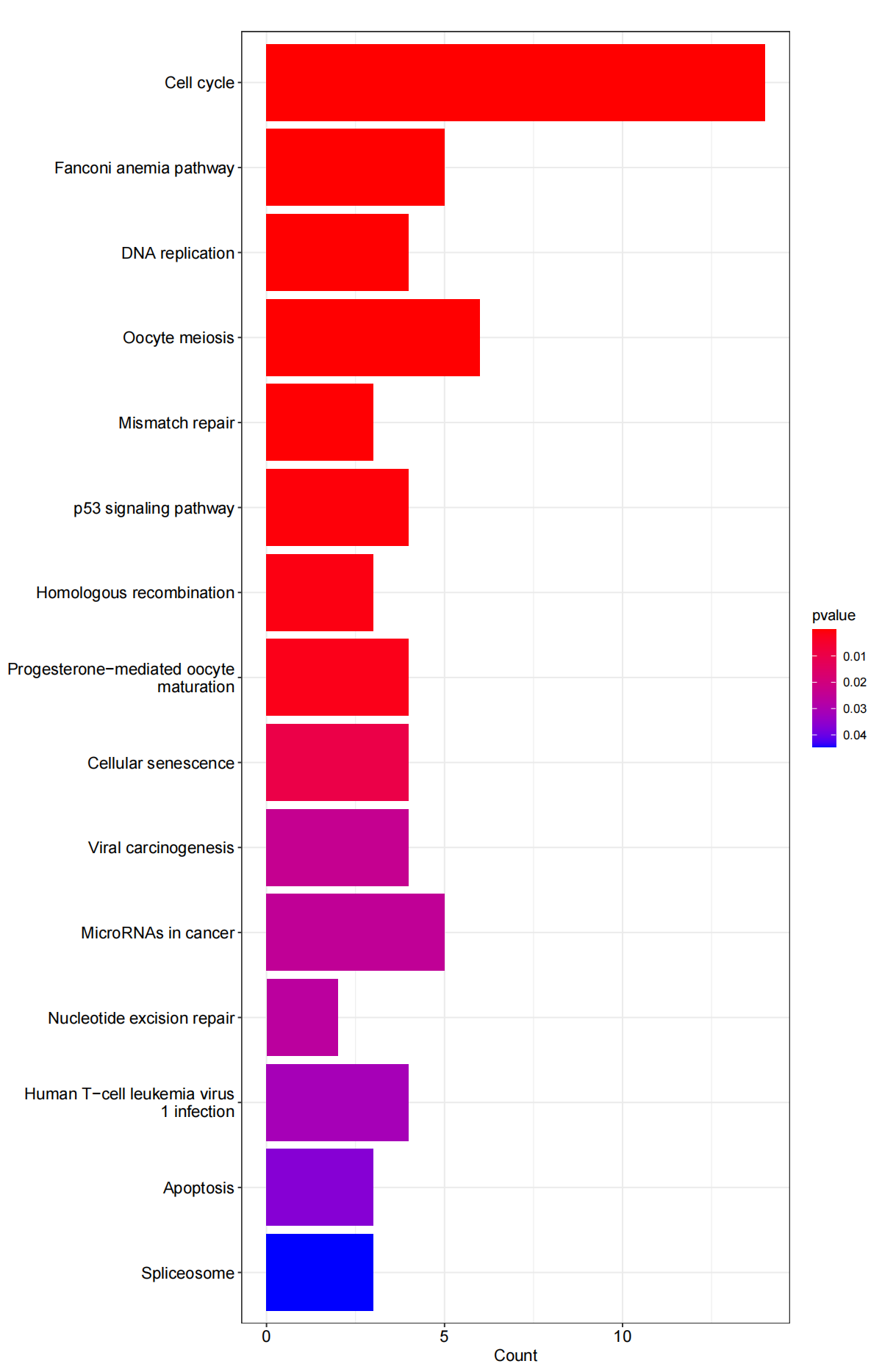

Supplement: Supplementary file 1 — Supplementary information. [file jcav15p1328s1.zip › Images Based on Data Mining and Bioinformatics Methods/kegg.png]

# HAUS1

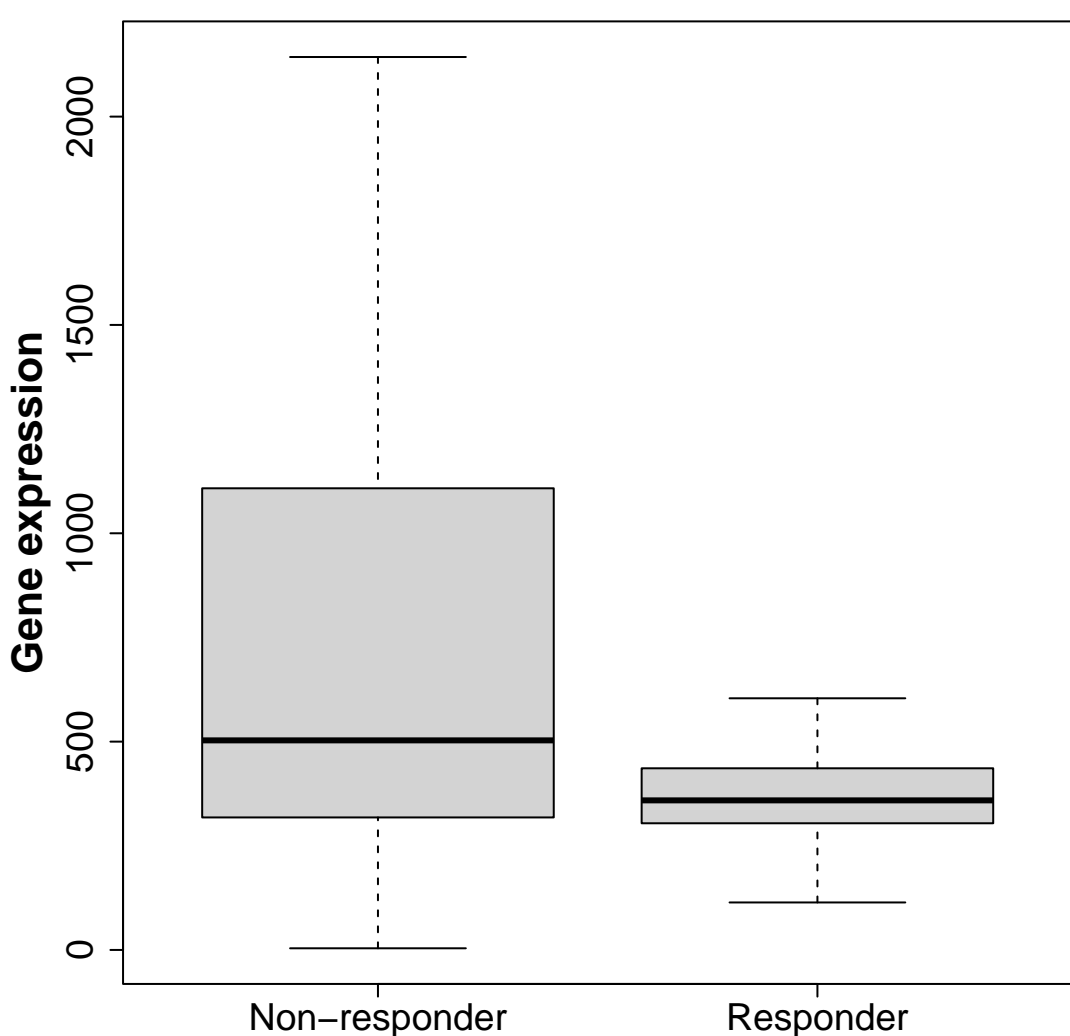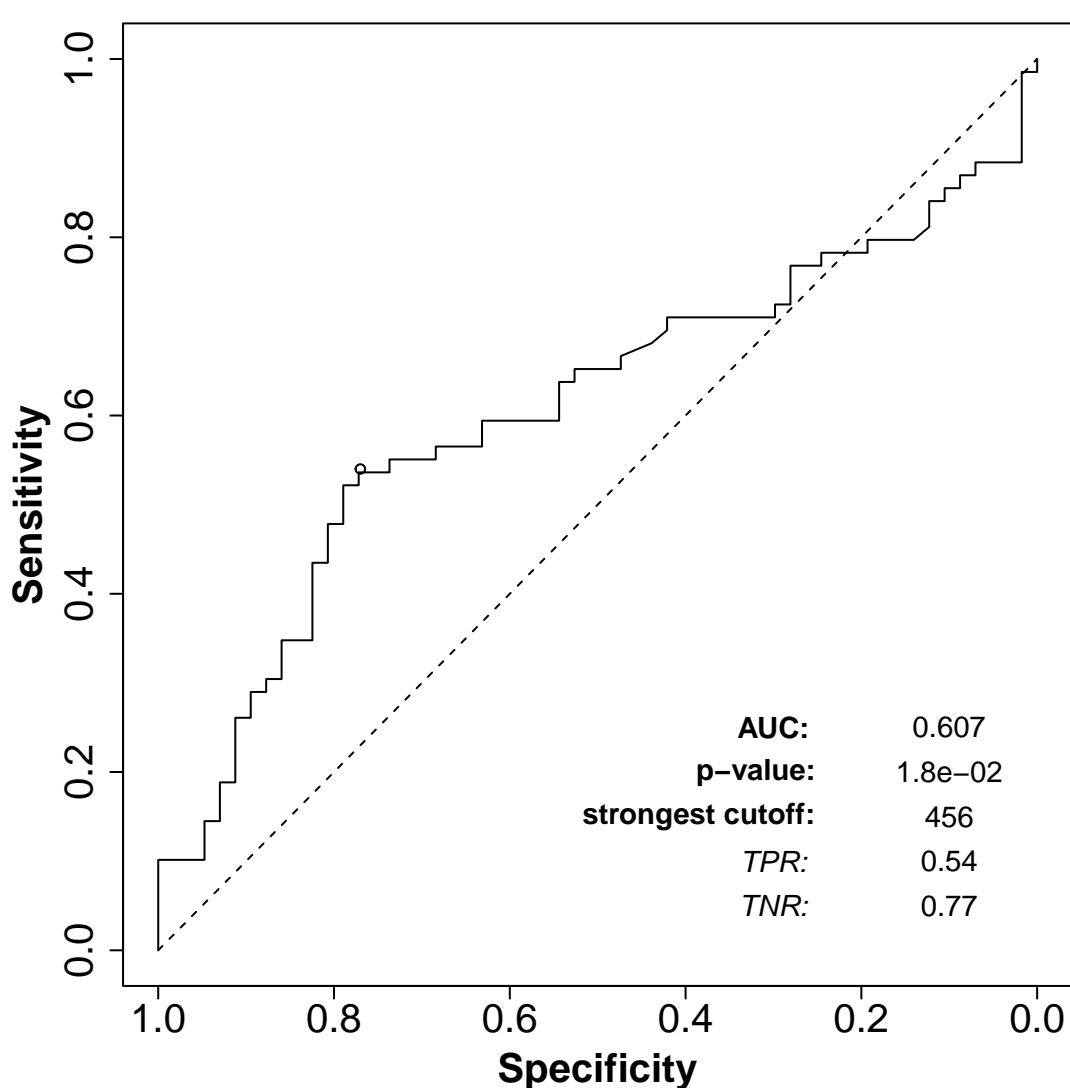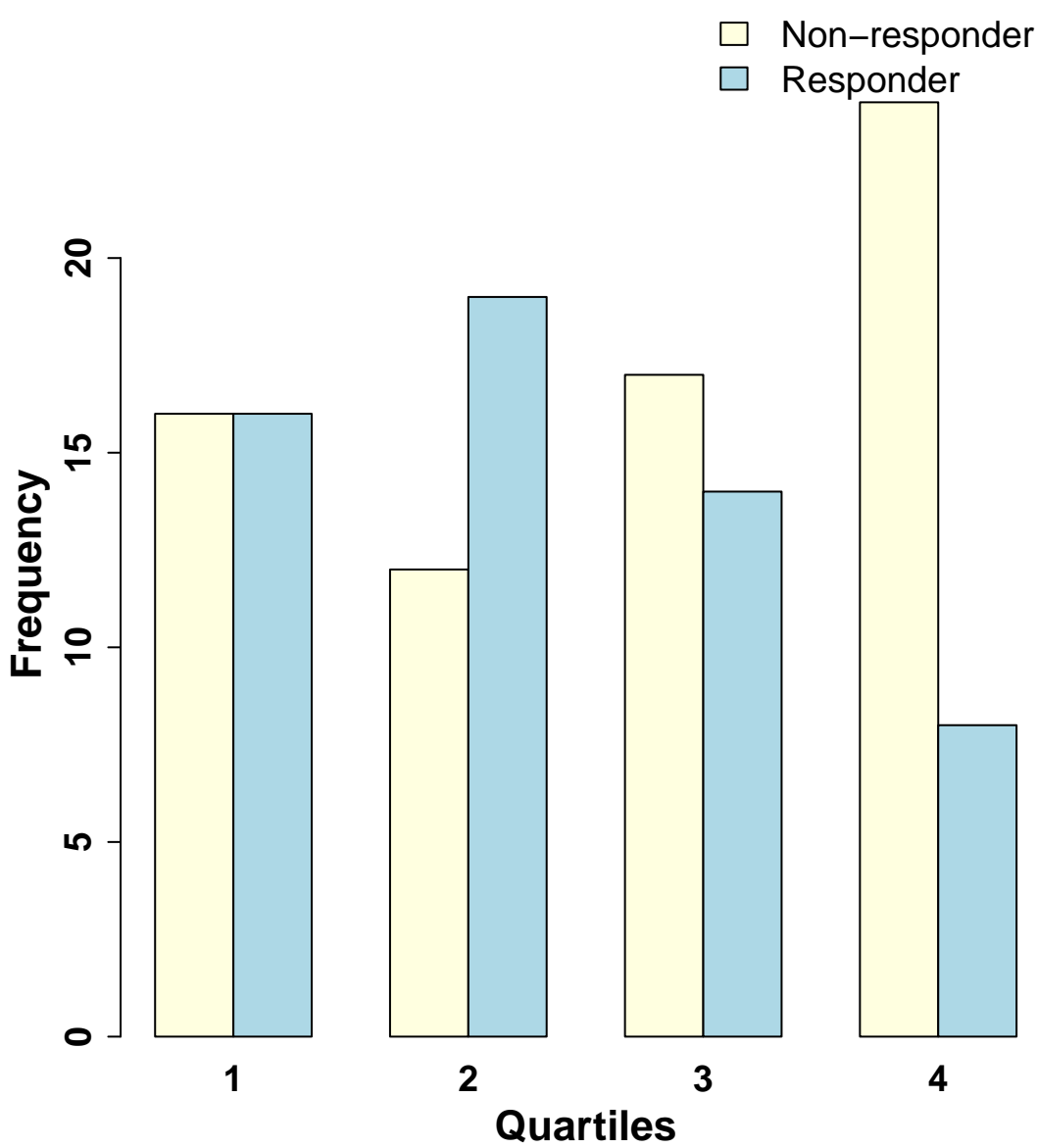

Supplement: Supplementary file 1 — Supplementary information. [file jcav15p1328s1.zip › Images Based on Data Mining and Bioinformatics Methods/KM/CTLA4 (CD152).pdf]

# DSS with the expression of HAUS1

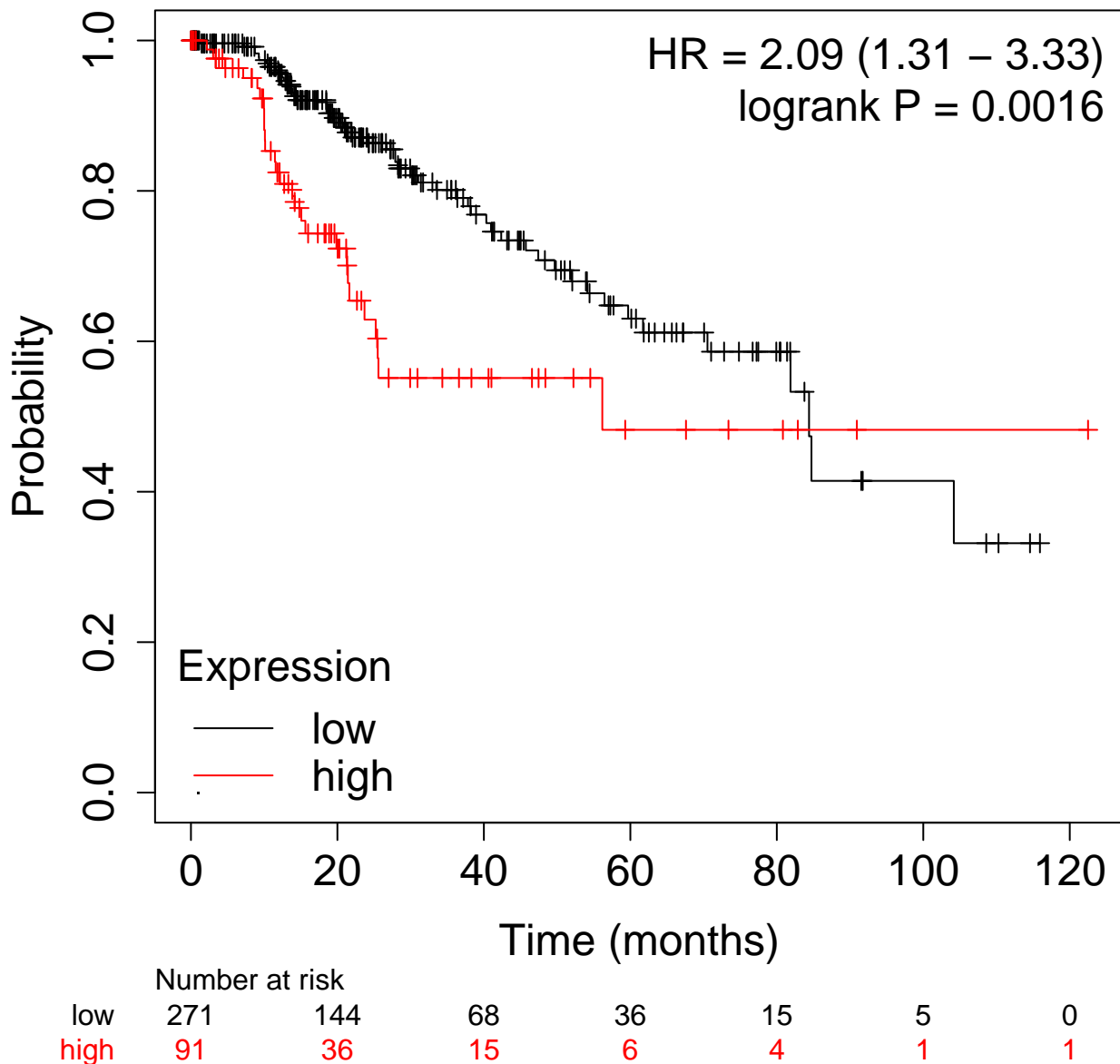

Supplement: Supplementary file 1 — Supplementary information. [file jcav15p1328s1.zip › Images Based on Data Mining and Bioinformatics Methods/KM/DSS1.pdf]

# OS with the expression of HAUS1

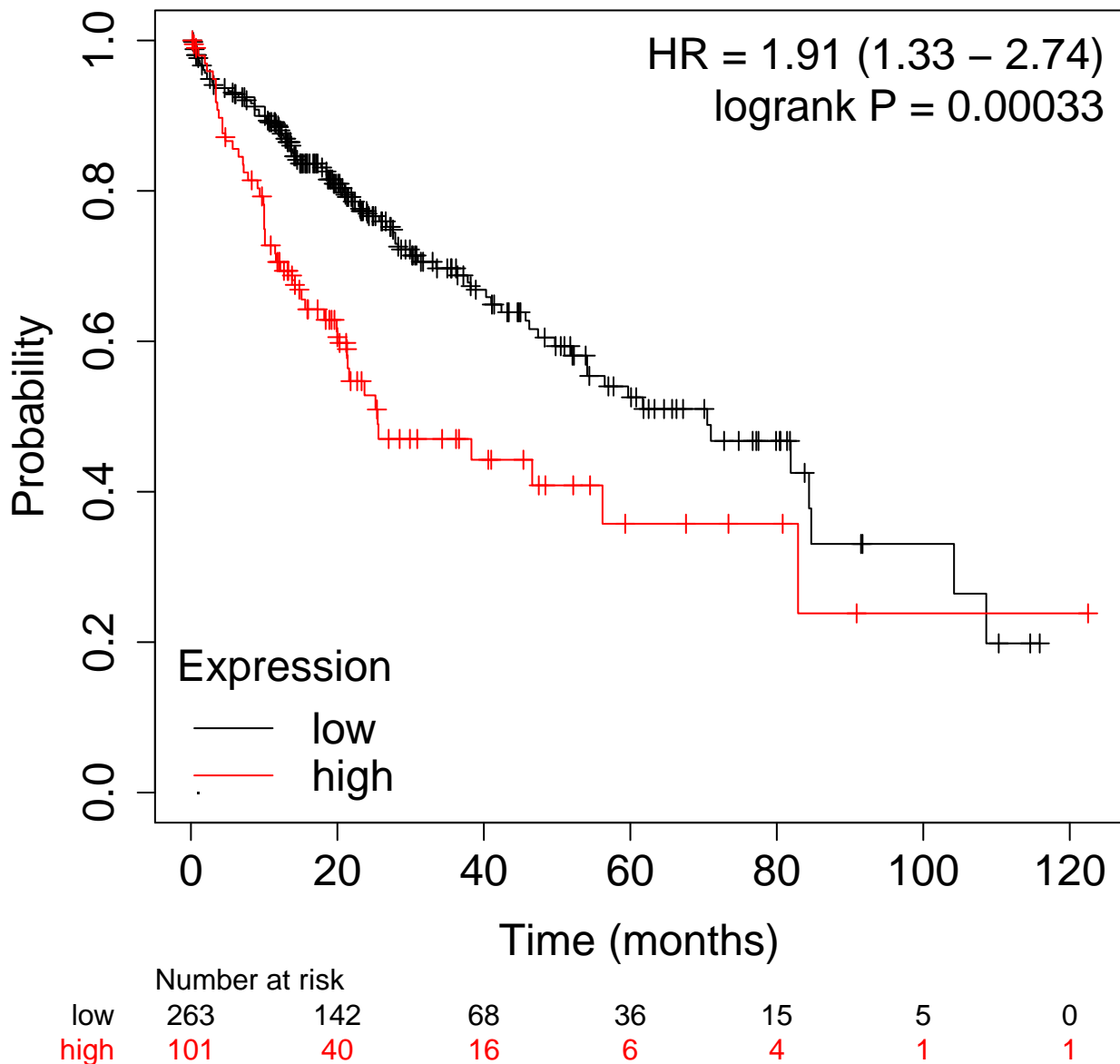

Supplement: Supplementary file 1 — Supplementary information. [file jcav15p1328s1.zip › Images Based on Data Mining and Bioinformatics Methods/KM/OS1.pdf]

# HAUS1

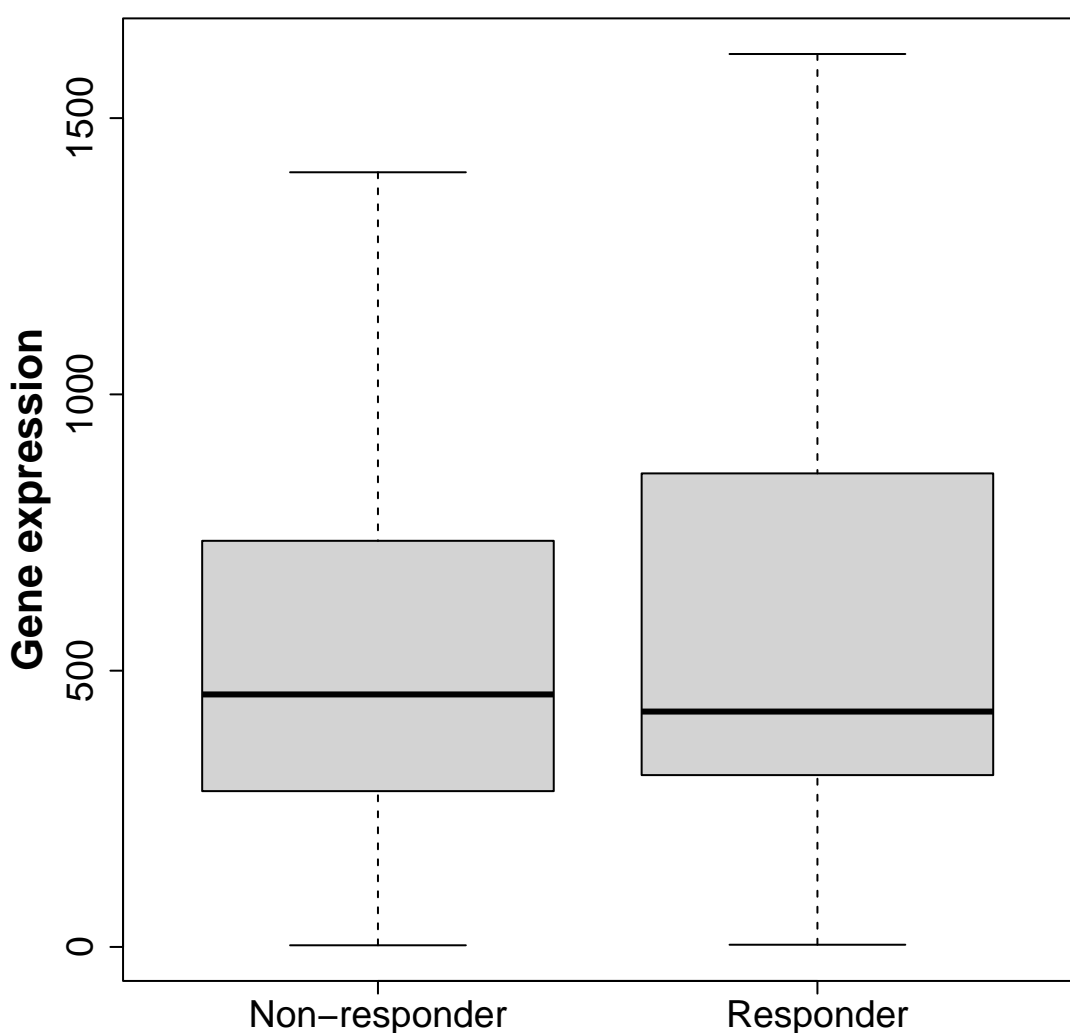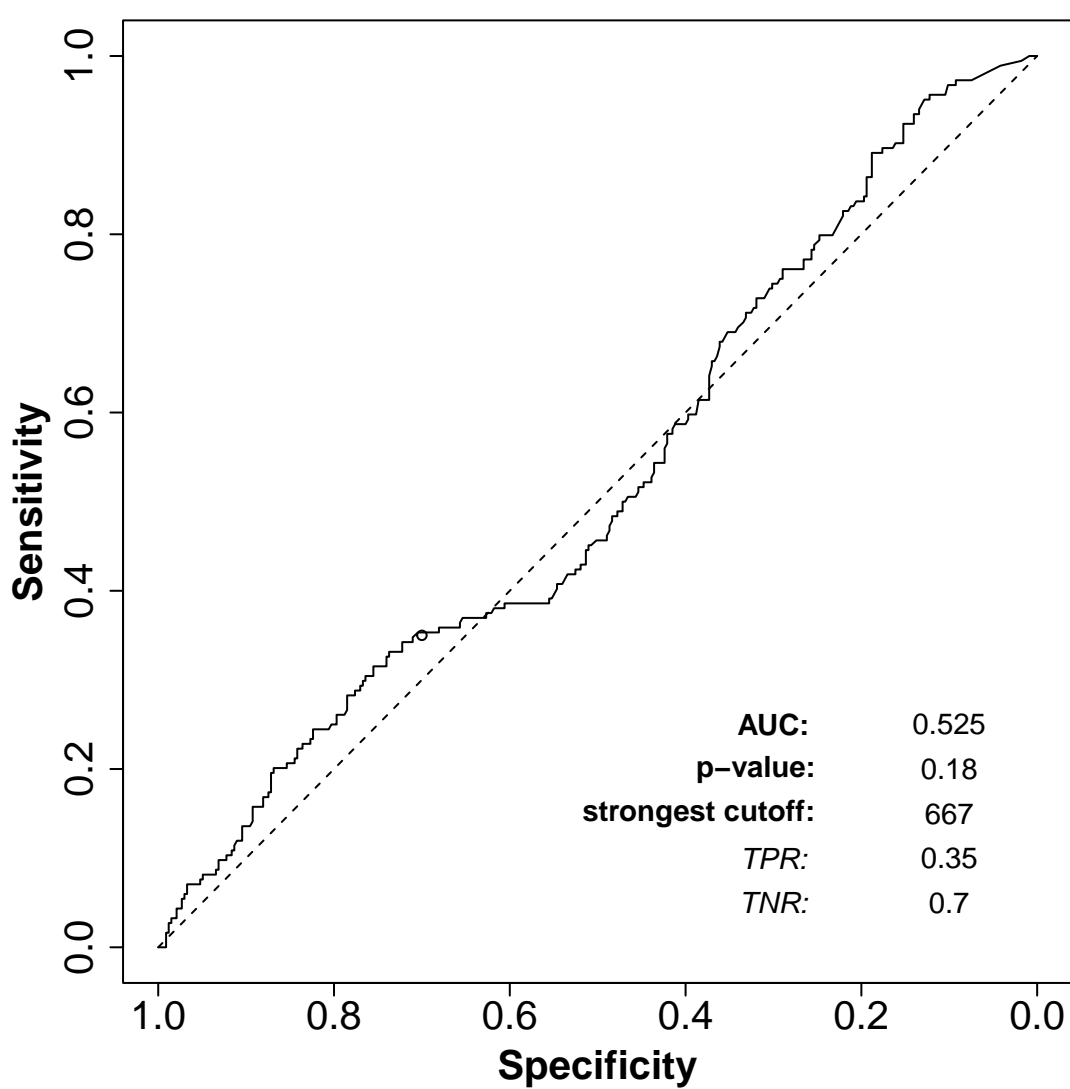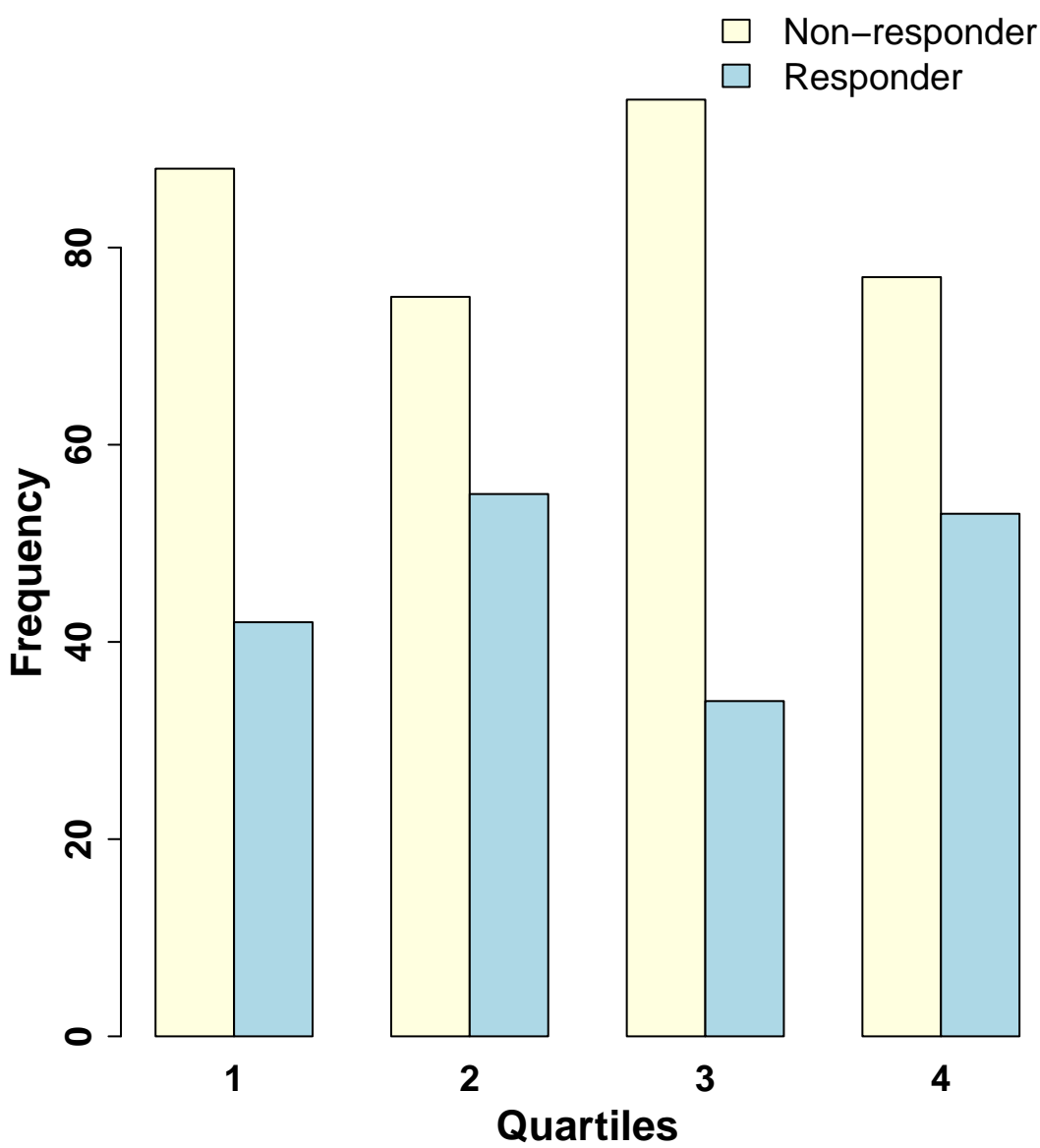

Supplement: Supplementary file 1 — Supplementary information. [file jcav15p1328s1.zip › Images Based on Data Mining and Bioinformatics Methods/KM/PD-1 (CD279) .pdf]

# HAUS1

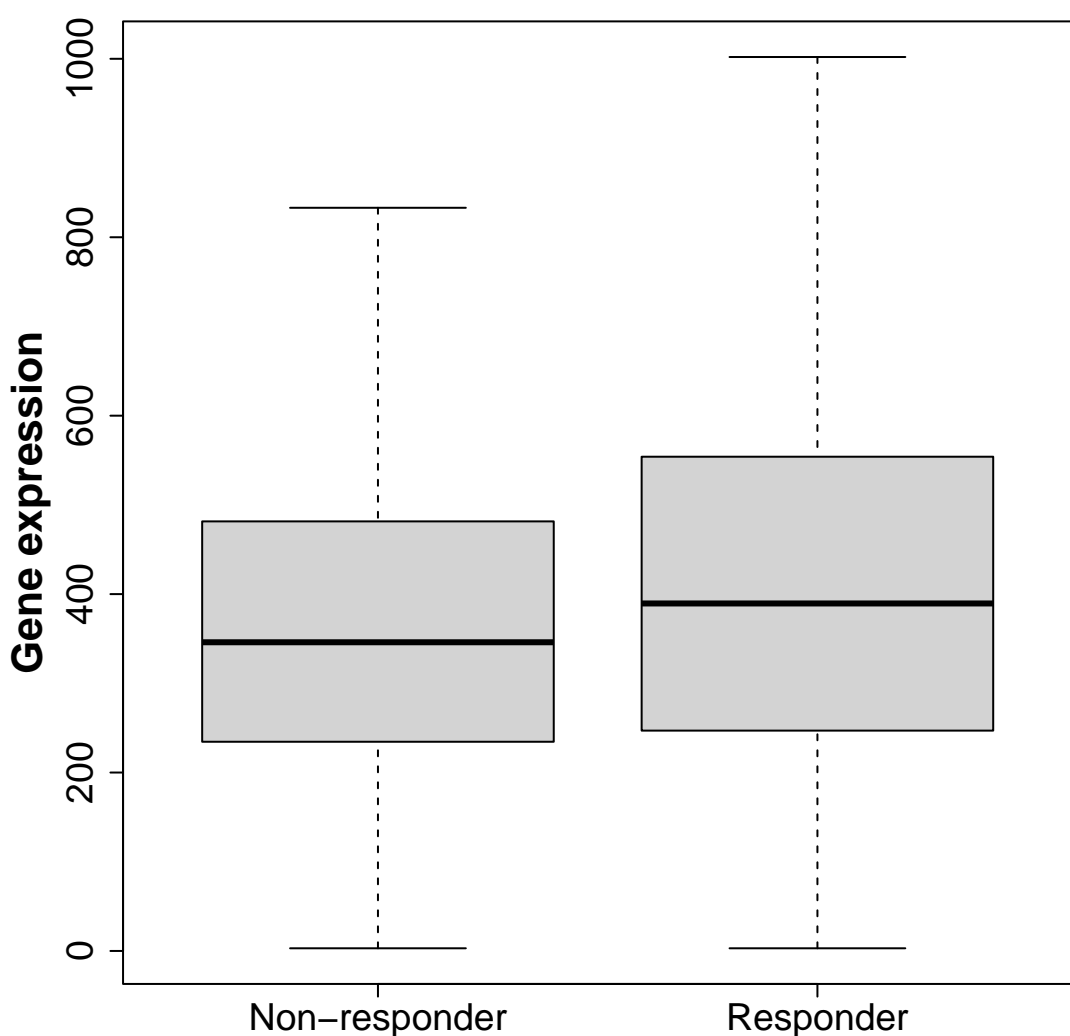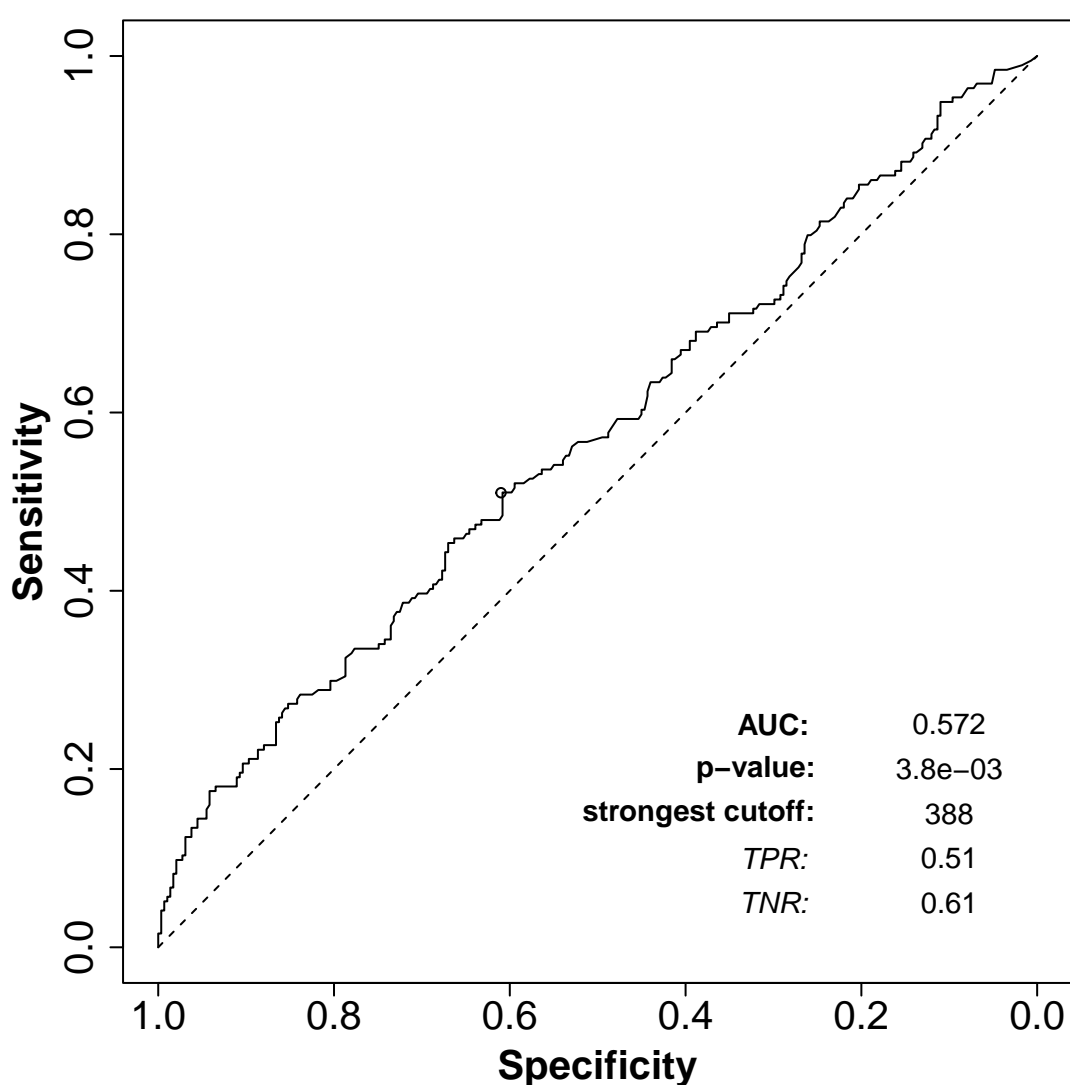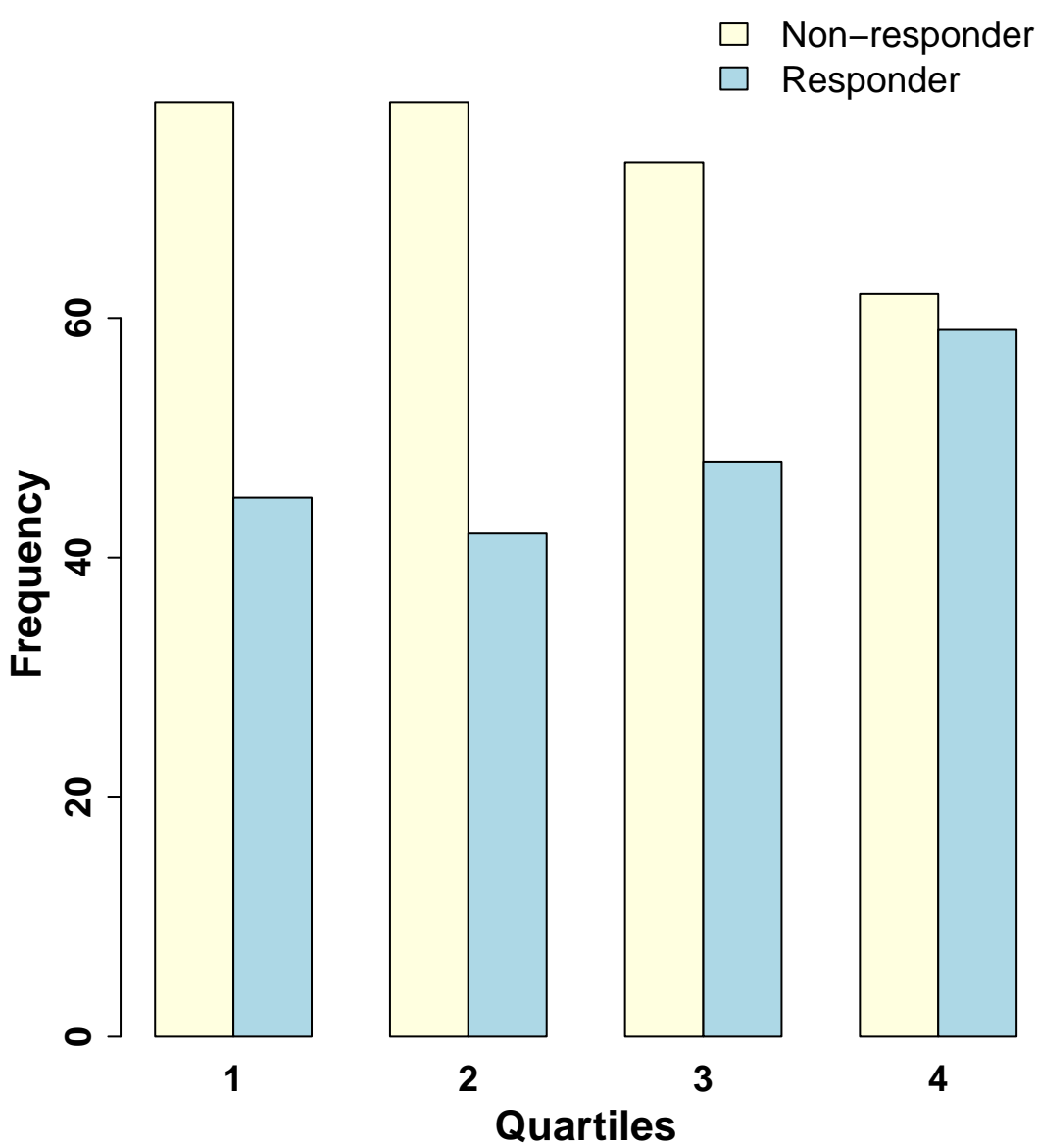

Supplement: Supplementary file 1 — Supplementary information. [file jcav15p1328s1.zip › Images Based on Data Mining and Bioinformatics Methods/KM/PD-L1 (CD274) .pdf]

# PFS with the expression of HAUS1

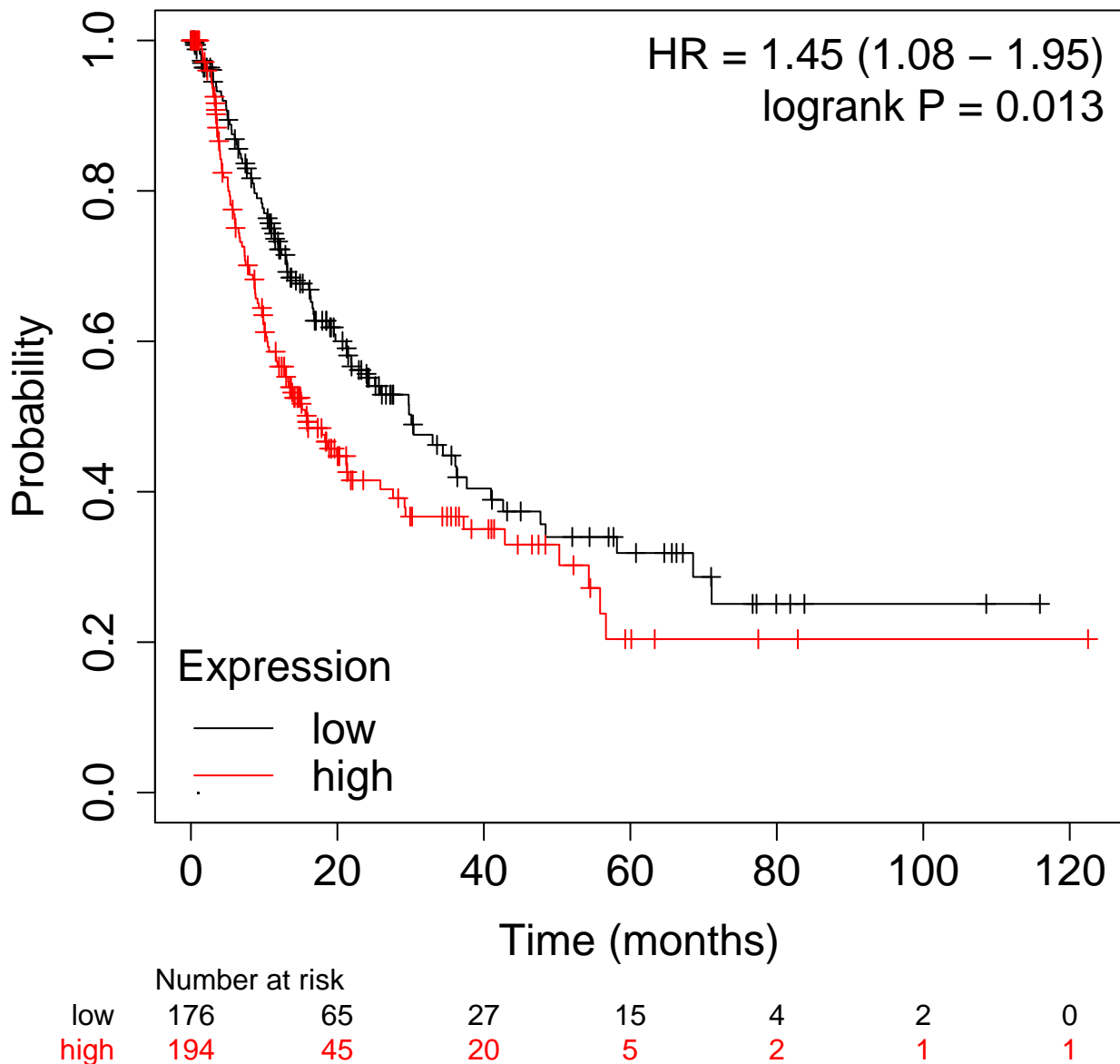

Supplement: Supplementary file 1 — Supplementary information. [file jcav15p1328s1.zip › Images Based on Data Mining and Bioinformatics Methods/KM/PFS1.pdf]

# RFS with the expression of HAUS1

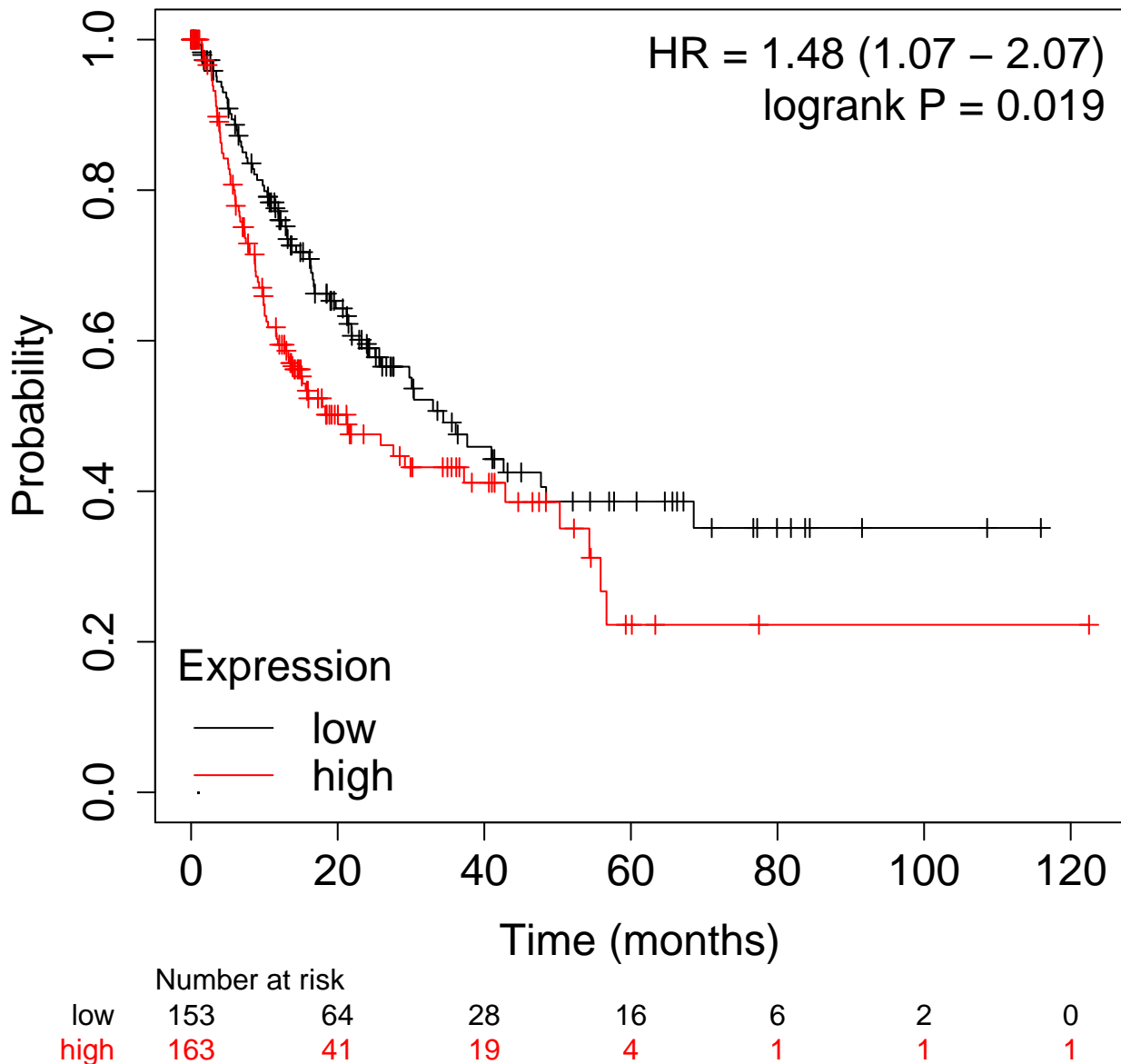

Supplement: Supplementary file 1 — Supplementary information. [file jcav15p1328s1.zip › Images Based on Data Mining and Bioinformatics Methods/KM/RFFS1.pdf]

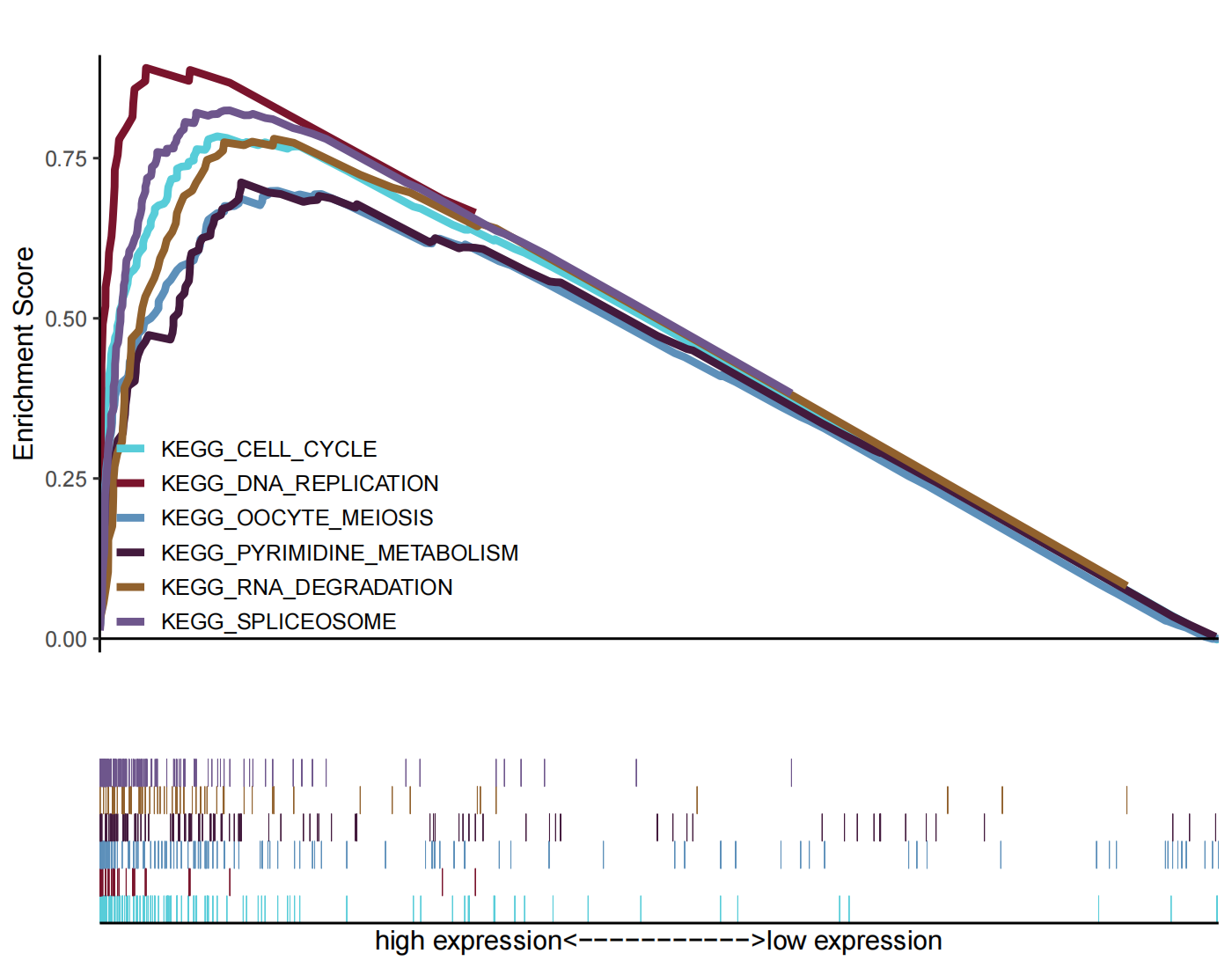

Supplement: Supplementary file 1 — Supplementary information. [file jcav15p1328s1.zip › Images Based on Data Mining and Bioinformatics Methods/multipleGSEA_00.png]

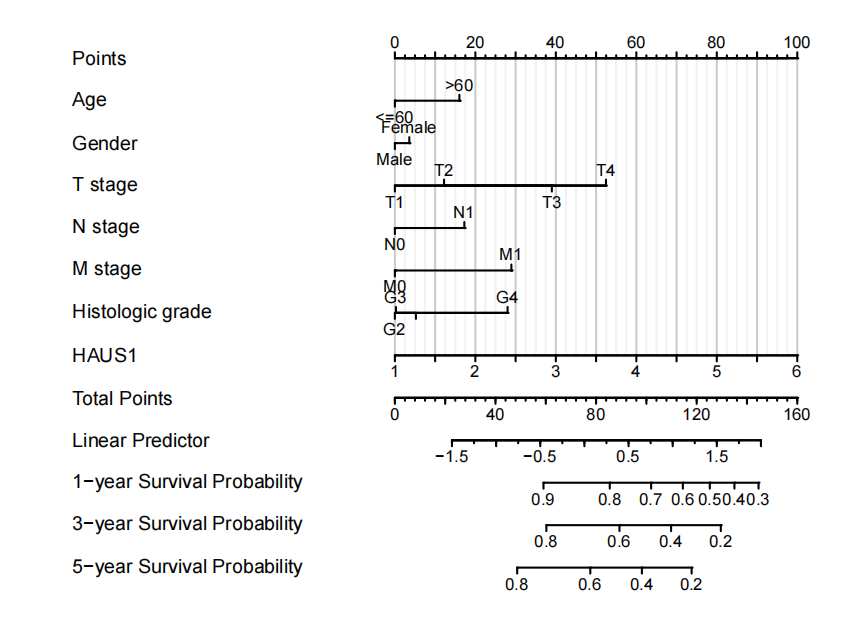

Supplement: Supplementary file 1 — Supplementary information. [file jcav15p1328s1.zip › Images Based on Data Mining and Bioinformatics Methods/Nomogram图_2022-07-11_10_40_35_00.png]

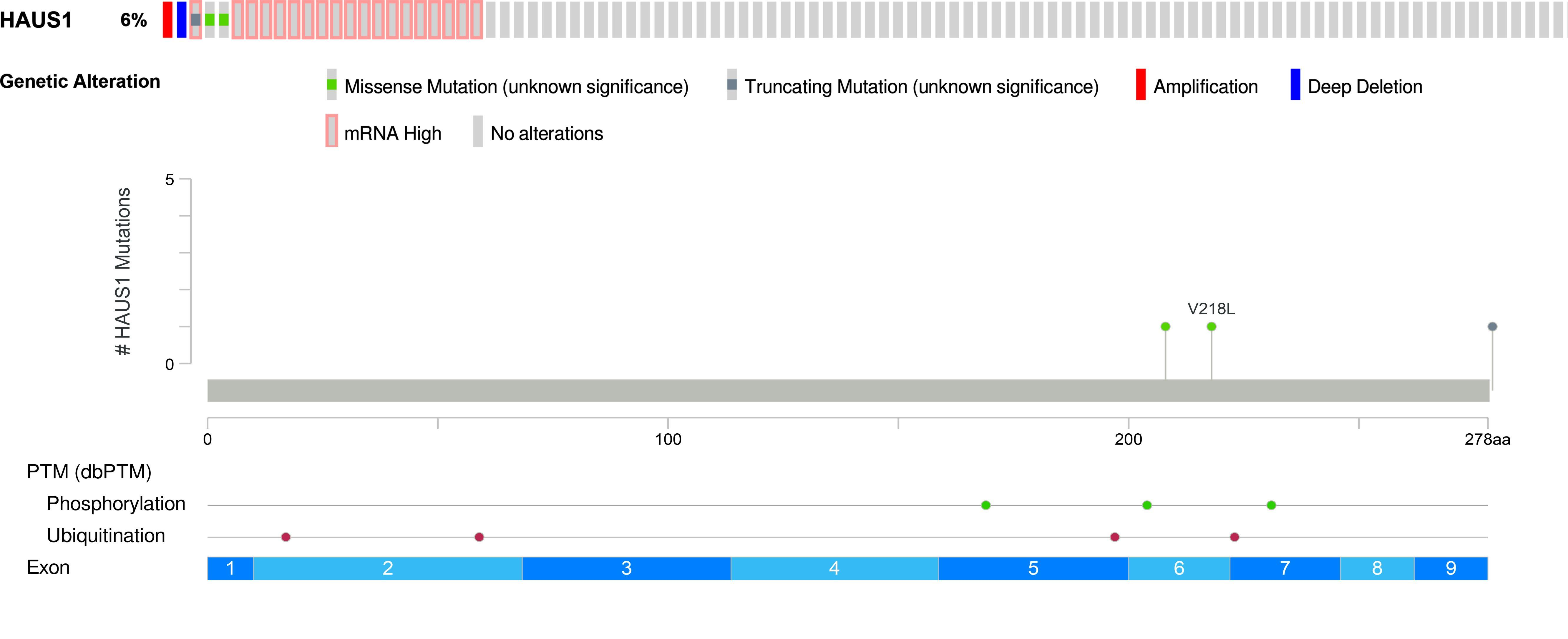

Supplement: Supplementary file 1 — Supplementary information. [file jcav15p1328s1.zip › Images Based on Data Mining and Bioinformatics Methods/oncoprint(1).png]

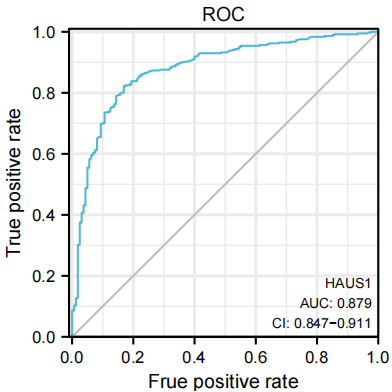

Supplement: Supplementary file 1 — Supplementary information. [file jcav15p1328s1.zip › Images Based on Data Mining and Bioinformatics Methods/ROC曲线_2022-10-23_21_44_26_00.png]

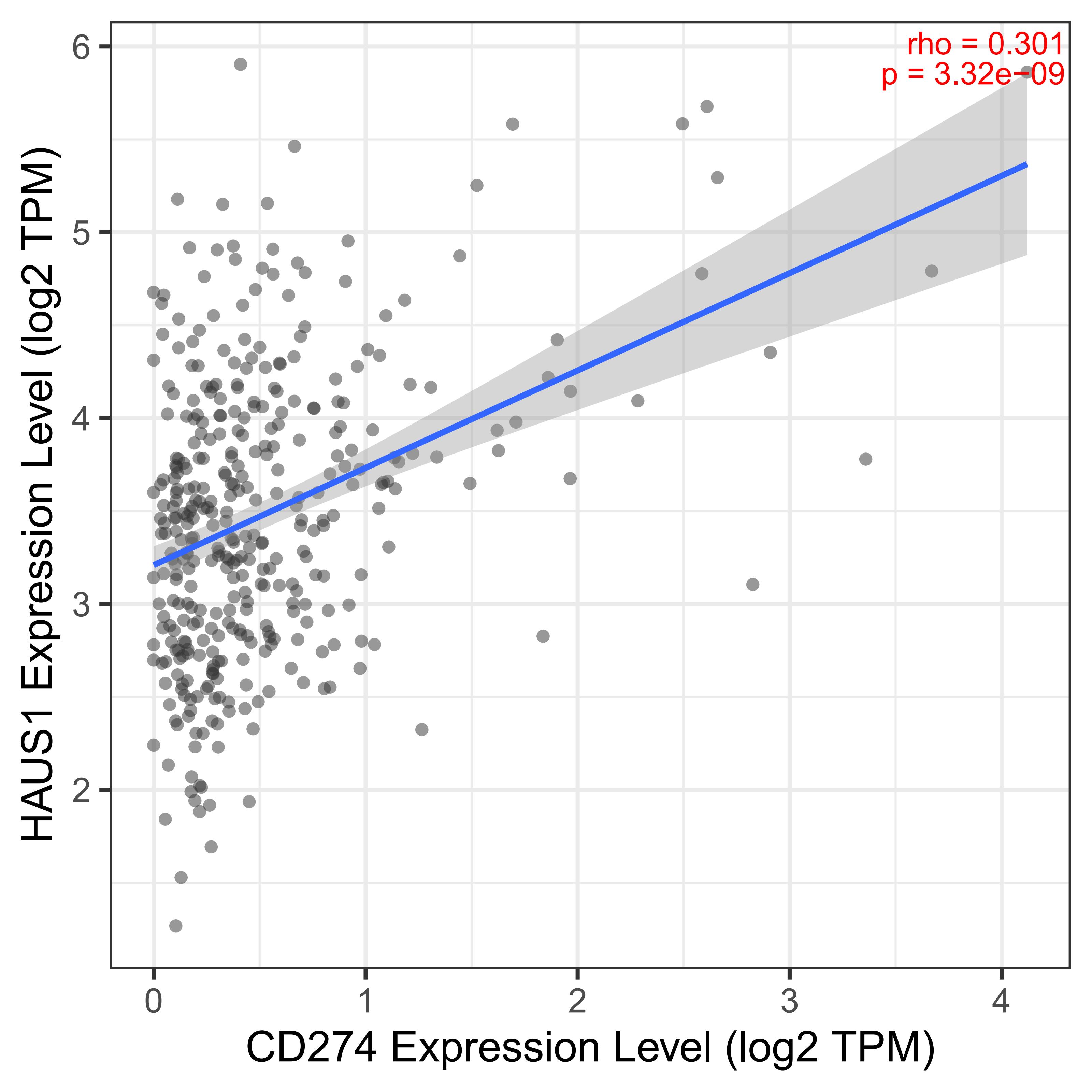

Supplement: Supplementary file 1 — Supplementary information. [file jcav15p1328s1.zip › Images Based on Data Mining and Bioinformatics Methods/TIMER2.0/CD274_00(1).jpg]

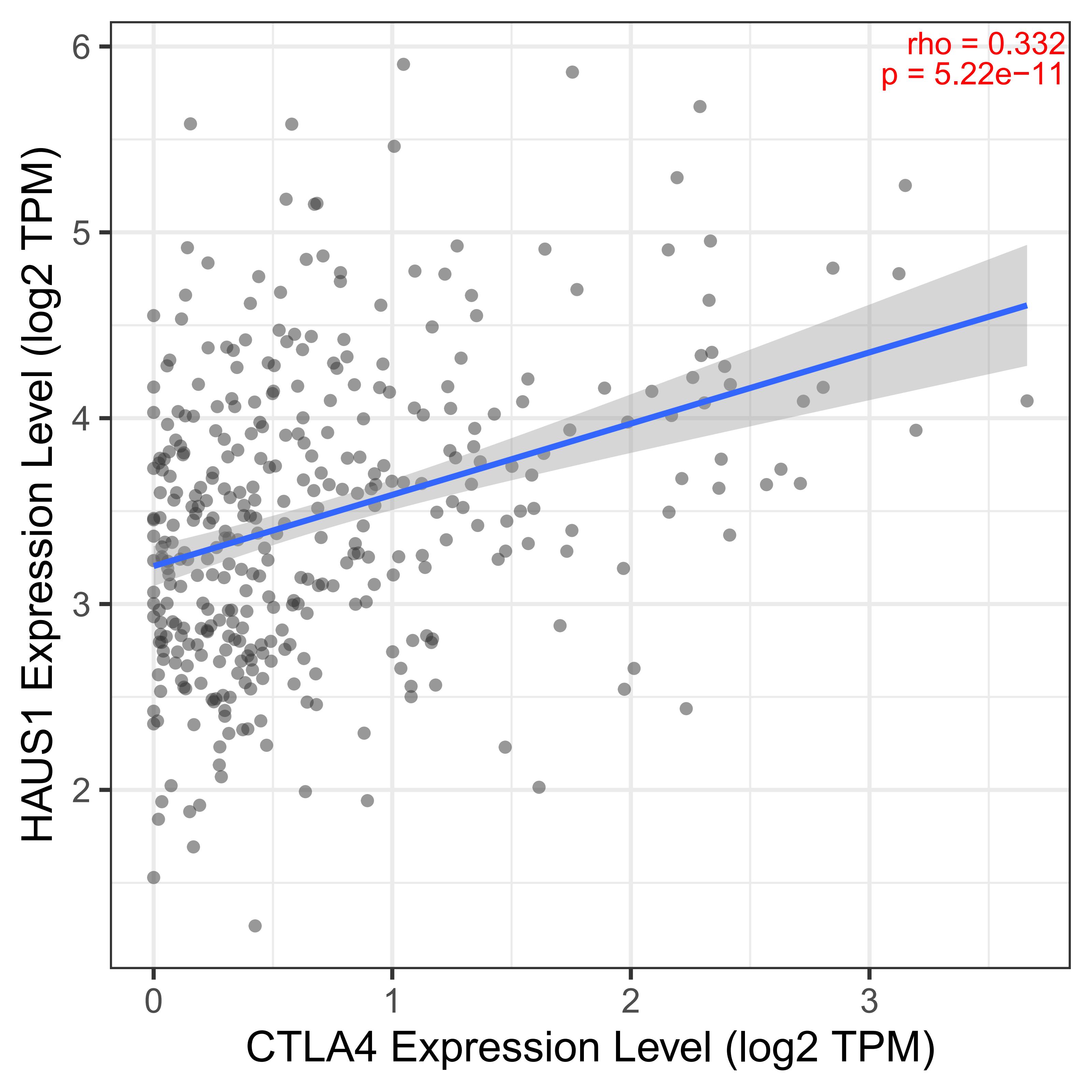

Supplement: Supplementary file 1 — Supplementary information. [file jcav15p1328s1.zip › Images Based on Data Mining and Bioinformatics Methods/TIMER2.0/CTLA4_00.jpg]

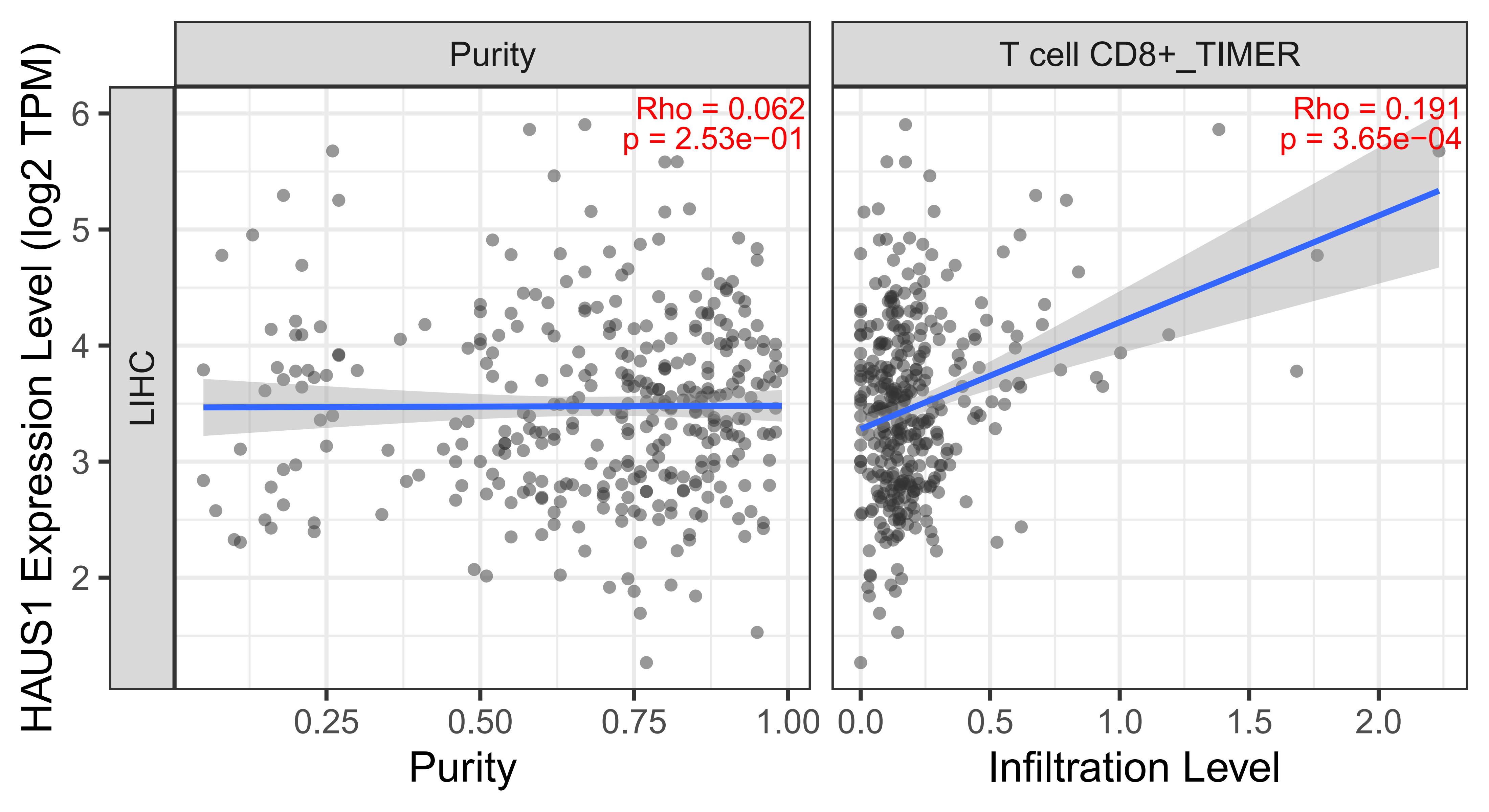

Supplement: Supplementary file 1 — Supplementary information. [file jcav15p1328s1.zip › Images Based on Data Mining and Bioinformatics Methods/TIMER2.0/gene_plot (1)_00.jpg]

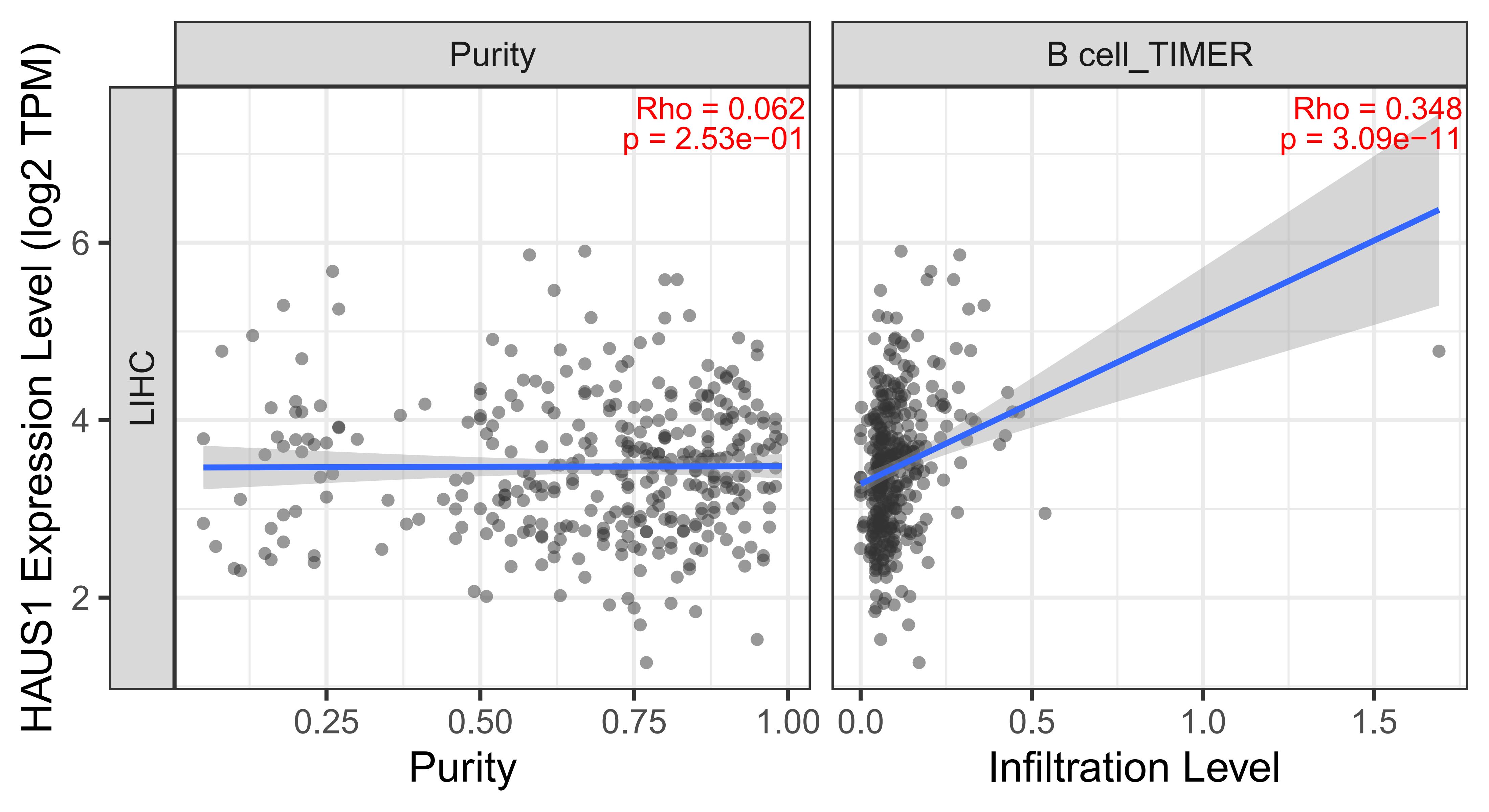

Supplement: Supplementary file 1 — Supplementary information. [file jcav15p1328s1.zip › Images Based on Data Mining and Bioinformatics Methods/TIMER2.0/gene_plot (2)_00.jpg]

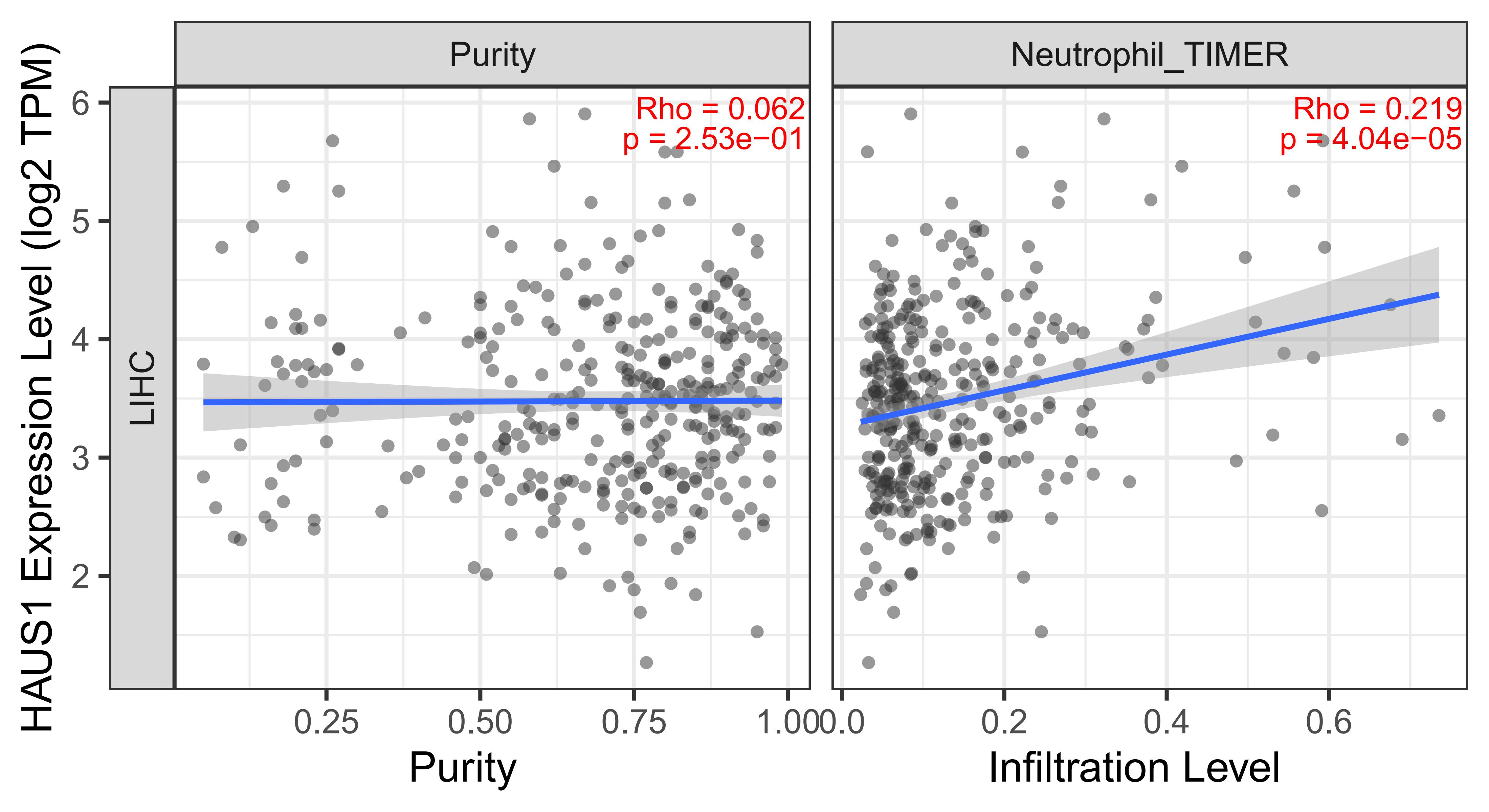

Supplement: Supplementary file 1 — Supplementary information. [file jcav15p1328s1.zip › Images Based on Data Mining and Bioinformatics Methods/TIMER2.0/gene_plot (3)_00.jpg]

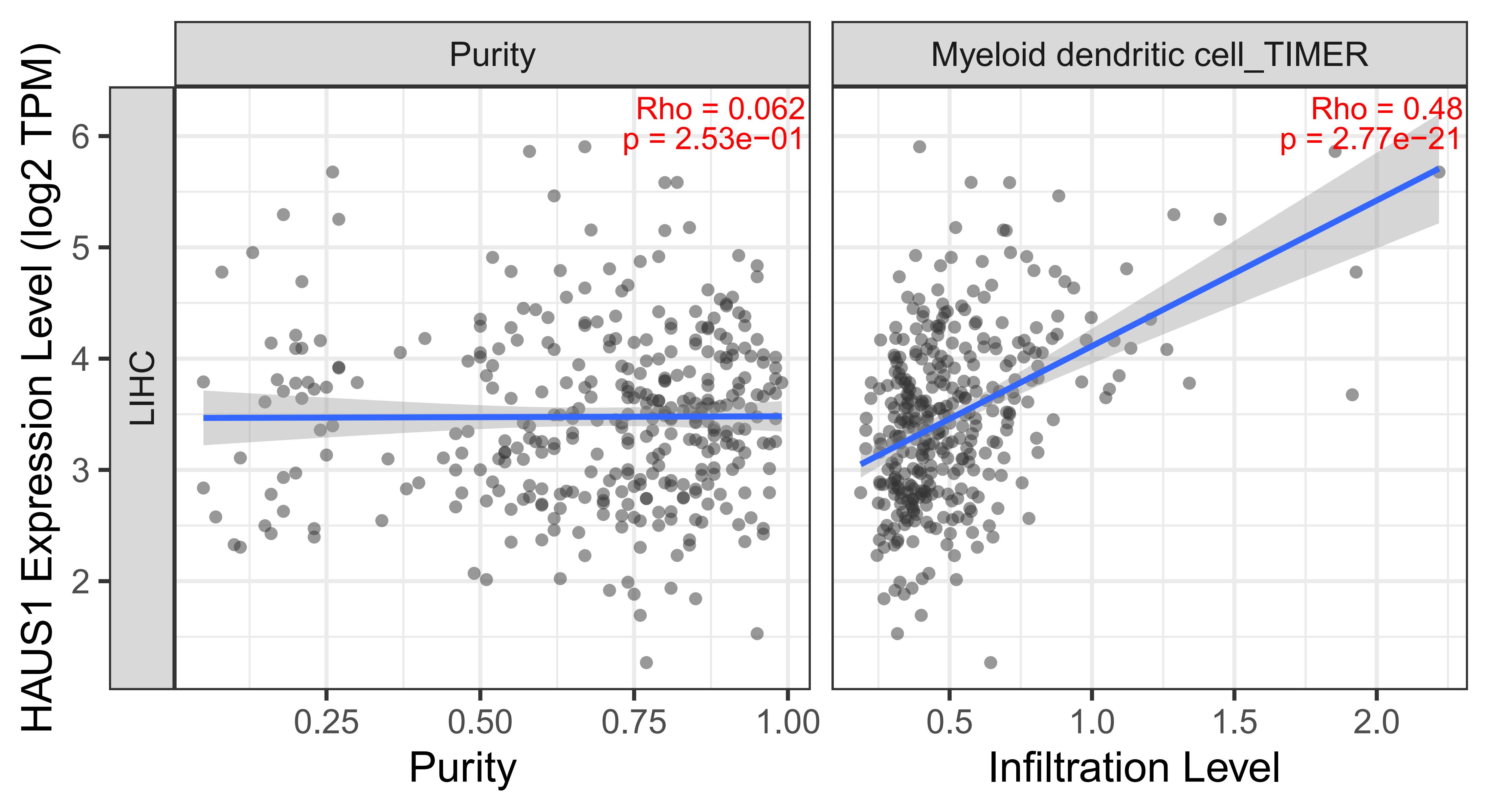

Supplement: Supplementary file 1 — Supplementary information. [file jcav15p1328s1.zip › Images Based on Data Mining and Bioinformatics Methods/TIMER2.0/gene_plot (6)_00.jpg]

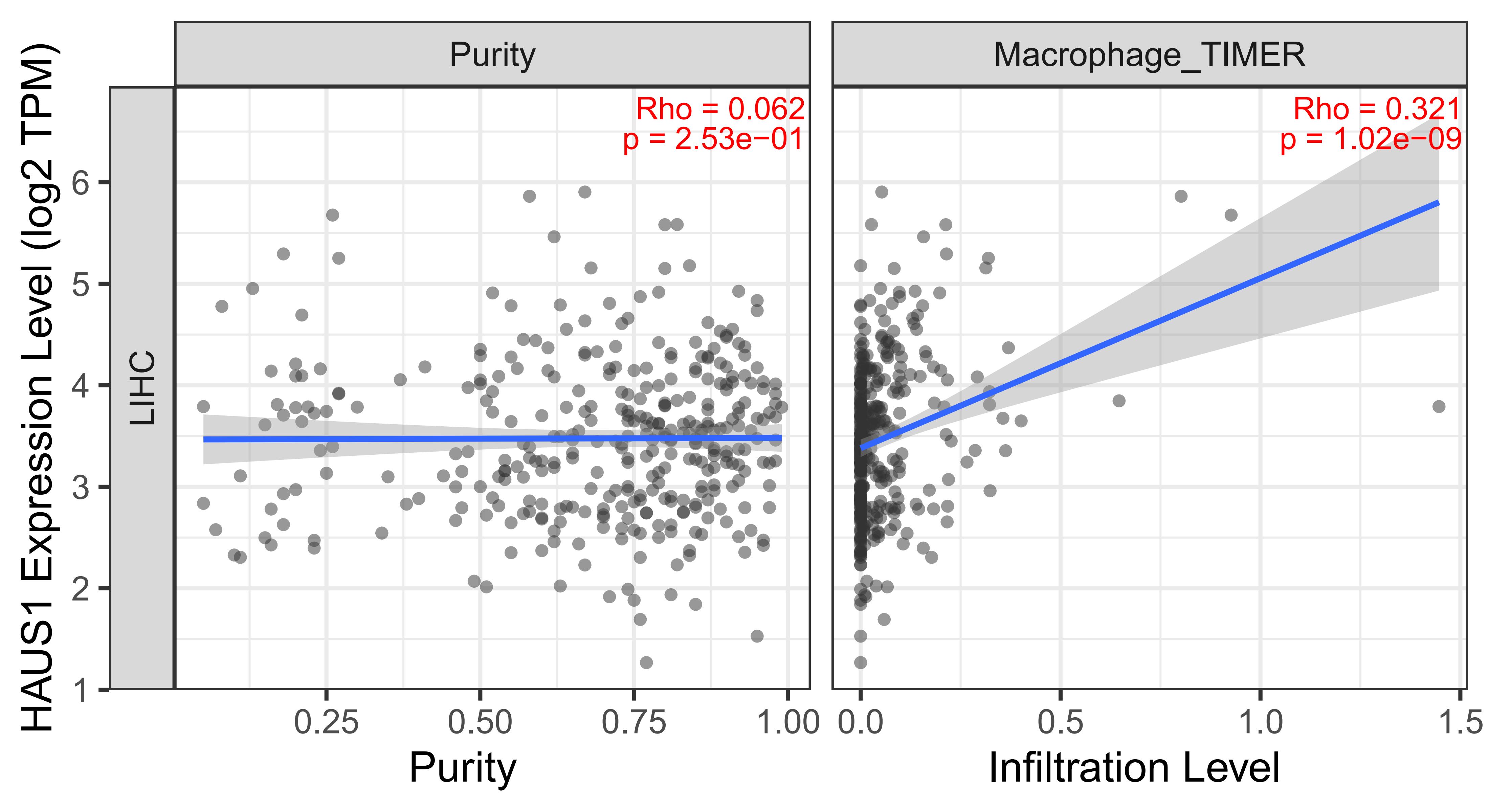

Supplement: Supplementary file 1 — Supplementary information. [file jcav15p1328s1.zip › Images Based on Data Mining and Bioinformatics Methods/TIMER2.0/gene_plot4_00.jpg]

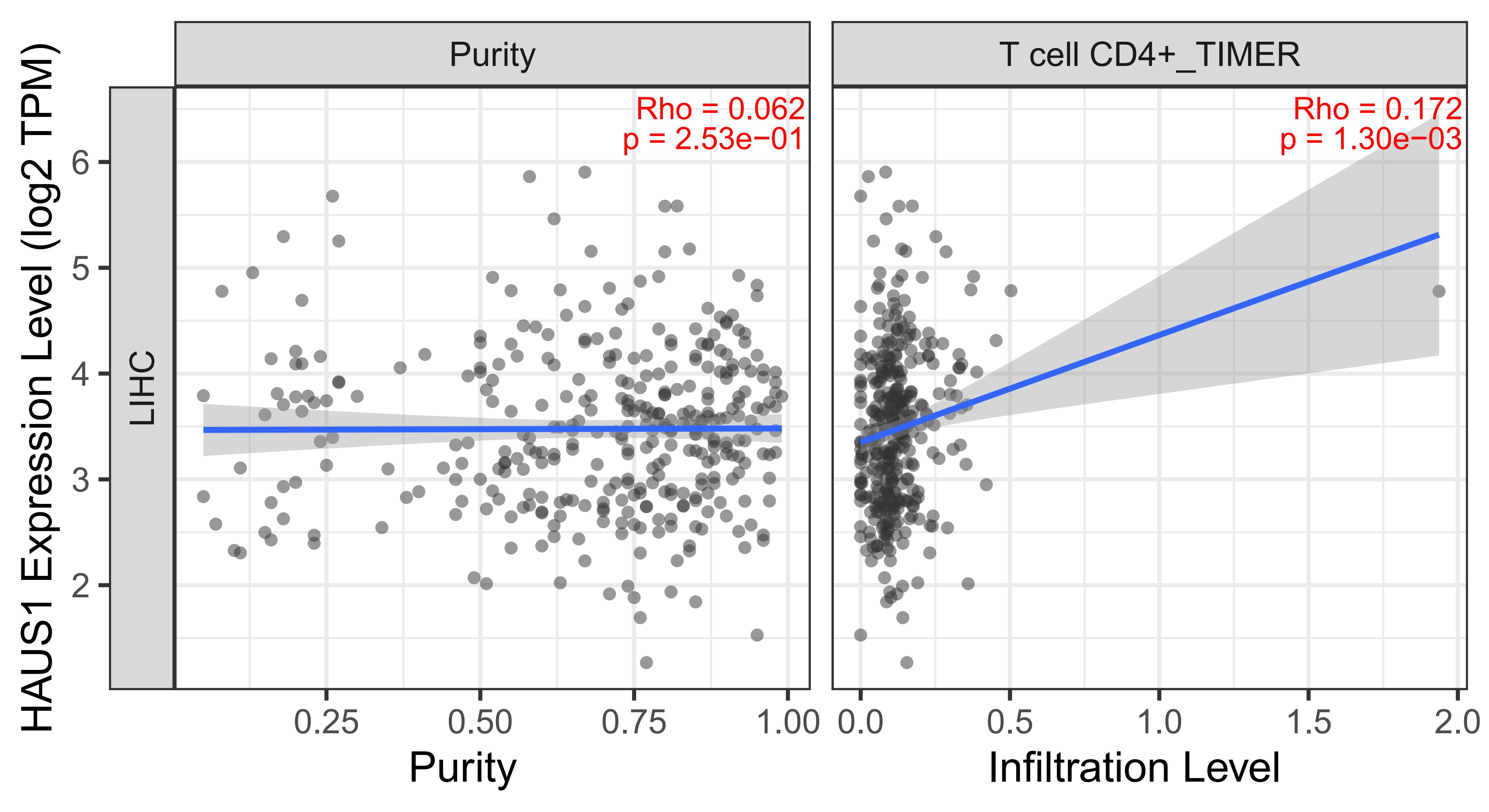

Supplement: Supplementary file 1 — Supplementary information. [file jcav15p1328s1.zip › Images Based on Data Mining and Bioinformatics Methods/TIMER2.0/gene_plot_00.jpg]

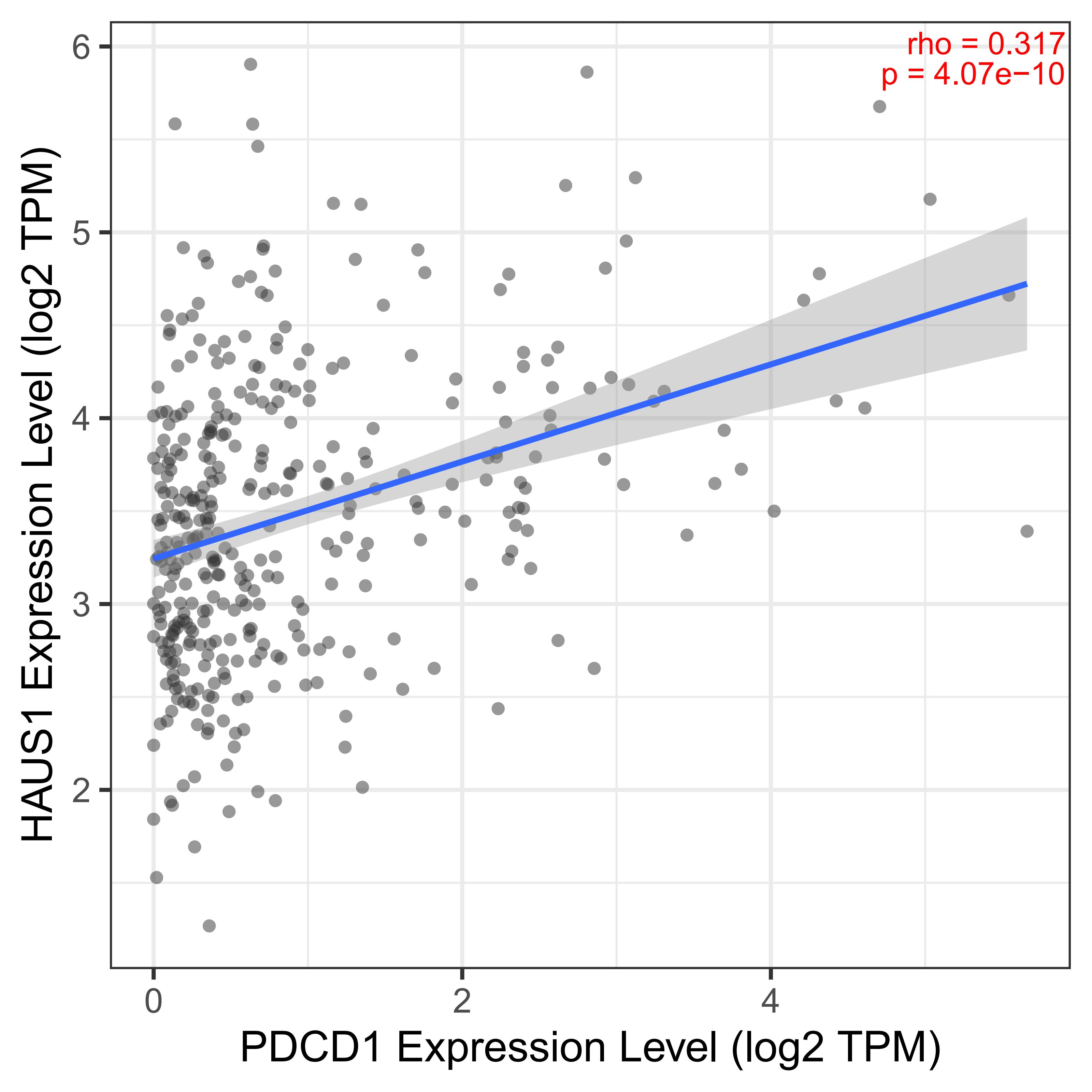

Supplement: Supplementary file 1 — Supplementary information. [file jcav15p1328s1.zip › Images Based on Data Mining and Bioinformatics Methods/TIMER2.0/PDCD1_00.jpg]

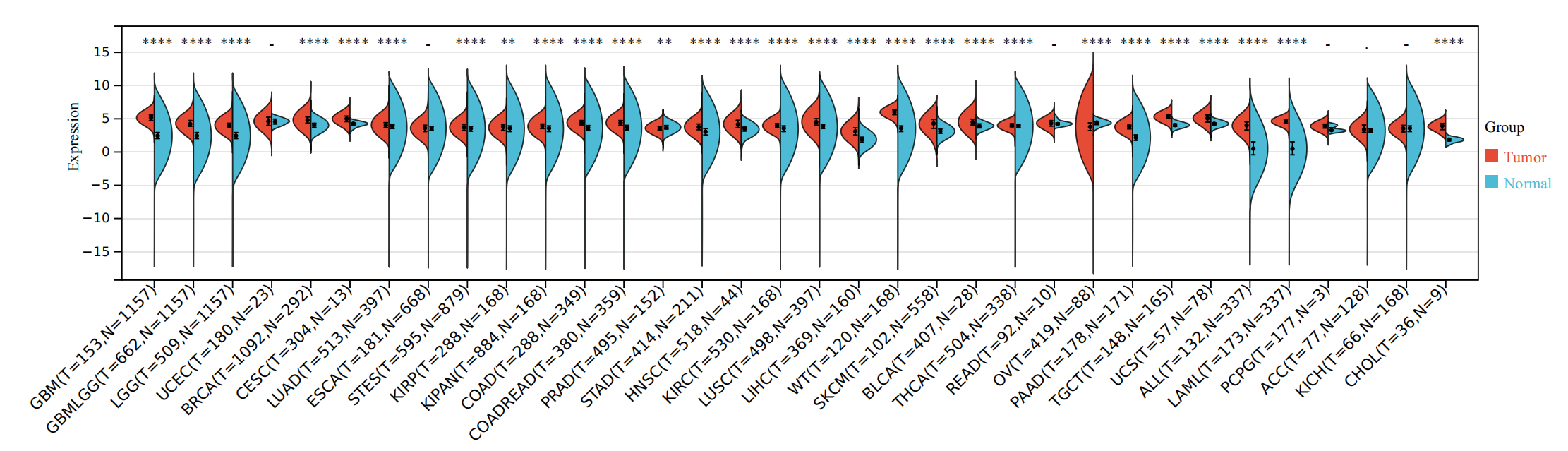

Supplement: Supplementary file 1 — Supplementary information. [file jcav15p1328s1.zip › Images Based on Data Mining and Bioinformatics Methods/基因表达差异_00.png]
